# Serine 363 of a Hydrophobic Region of Archaeal Ribulose 1,5-Bisphosphate Carboxylase/Oxygenase from *Archaeoglobus fulgidus* and *Thermococcus kodakaraensis* Affects CO_2_/O_2_ Substrate Specificity and Oxygen Sensitivity

**DOI:** 10.1371/journal.pone.0138351

**Published:** 2015-09-18

**Authors:** Nathan E. Kreel, F. Robert Tabita

**Affiliations:** 1 The Ohio State University Biochemistry Program, The Ohio State University, 484 West 12th Avenue, Columbus, Ohio, 43210–1292, United States of America; 2 Department of Microbiology, The Ohio State University, 484 West 12th Avenue, Columbus, Ohio, 43210–1292, United States of America; University of Freiburg, GERMANY

## Abstract

Archaeal ribulose 1, 5-bisphospate carboxylase/oxygenase (RubisCO) is differentiated from other RubisCO enzymes and is classified as a form III enzyme, as opposed to the form I and form II RubisCOs typical of chemoautotrophic bacteria and prokaryotic and eukaryotic phototrophs. The form III enzyme from archaea is particularly interesting as several of these proteins exhibit unusual and reversible sensitivity to molecular oxygen, including the enzyme from *Archaeoglobus fulgidus*. Previous studies with *A*. *fulgidus* RbcL2 had shown the importance of Met-295 in oxygen sensitivity and pointed towards the potential significance of another residue (Ser-363) found in a hydrophobic pocket that is conserved in all RubisCO proteins. In the current study, further structure/function studies have been performed focusing on Ser-363 of *A*. *fulgidus* RbcL2; various changes in this and other residues of the hydrophobic pocket point to and definitively establish the importance of Ser-363 with respect to interactions with oxygen. In addition, previous findings had indicated discrepant CO_2_/O_2_ specificity determinations of the *Thermococcus kodakaraensis* RubisCO, a close homolog of *A*. *fulgidus* RbcL2. It is shown here that the *T*. *kodakaraensis* enzyme exhibits a similar substrate specificity as the *A*. *fulgidus* enzyme and is also oxygen sensitive, with equivalent residues involved in oxygen interactions.

## Introduction

Many eukaryotic and prokaryotic organisms assimilate and reduce carbon dioxide as sole carbon source via the Calvin-Benson-Bassham (CBB) reductive pentose phosphate pathway, with the key catalyst being ribulose-1,5-bisphosphate (RuBP) carboxylase/oxygenase (RubisCO) [1]. RubisCO is also able to use O_2_ as a gaseous substrate, leading to the formation of important and unique metabolic products that have deep physiological significance [1, 2–5]. Based on amino acid sequences, there are three types or clades of RubisCO proteins found in nature that catalyze the carboxylation or oxygenation of RuBP, forms I, II, and III [1, 4–7]. RubisCO is thought to be the most abundant enzyme on the planet as roughly 50–60% of plant leaf protein and autotrophic microbial biomass may be composed of this protein. Thus, RubisCO is theorized to be one of the major determinants for insuring the existence of human life on earth [8]. As such, recent studies on form I catalytic efficiency [9] and elucidation of factors involved in form I RubisCO assembly [10] have greatly advanced the possibilities that more effective catalysts might be constructed for enhanced physiological function.

The RubisCO catalytic mechanism is well understood [11] and the ability of this enzyme from different organisms to differentiate between the two gaseous substrates (CO_2_ or O_2_) is defined by the enzyme’s substrate specificity factor (Ω), in which Ω = V_c_K_o_/V_o_K_c_, with K the Michaelis-Menten constant for the carboxylase (K_c_) or oxygenase (K_o_) reactions, and V the maximal velocities for the carboxylase (V_c_) or oxygenase (V_o_) reactions [3]. (V_max_/K_m_) reflect the catalytic efficiencies for the carboxylase (V_c_/K_c_) and oxygenase (V_o_/K_o_) reactions, respectively. The initial velocities for the carboxylase (v_c_) or oxygenase (v_o_) reactions are further defined *a*s v_c_/v_o_ = Ω ([CO_2_]/[O_2_]). Thus, Ω essentially describes the enzyme’s catalytic efficiency for either the carboxylase or oxygenase reactions at any particular gaseous substrate concentration. Form I RubisCOs have the highest Ω values but also exhibit the largest range of Ω values, from 20–240. Form II enzymes have much lower Ω values that range between 10–15 [1]. Form III enzymes from archaea are particularly interesting as these organisms use RubisCO for processes other than primary CO_2_ fixation [12–15]. Moreover, many of these archaeal RubisCOs are derived from strictly anaerobic organisms, and some tend to show unusual, but reversible, high sensitivity to molecular oxygen [16–18]; e.g., these enzymes lose carboxylase activity when exposed to and assayed in the presence of oxygen, even in the presence of extremely high levels of CO_2_ that normally out-compete the effects of oxygen with form I and form II enzymes [16,17]. Moreover, archaeal RubisCOs from extremophiles tend to sustain activity under unusual conditions, such as high salt concentrations or at extremely high or even low temperatures [6,16,18–21]. The response of the *A*. *fulgidus* RubisCO to molecular oxygen was clearly shown to be a classic competition with CO_2_ for the enediolate intermediate of the enzyme [17], as observed for all RubisCO proteins. However, what distinguished the *A*. *fulgidus* enzyme from other sources is the extremely high capacity of this enzyme to interact with molecular oxygen, with K_i_ (or K_O_) values of about 5 μM [17]. This K_i_ is nearly 3 orders of magnitude lower than typical form I or form II enzymes, as is the K_i_ for the recently described RubisCO from *Methanococcoides burtonii* [19], a curious protein that has both form II and form III characteristics [6,19]. Clearly, this high apparent affinity for molecular oxygen of the *A*. *fulgidus* enzyme accentuates the fact that carboxylase activity is completely inhibited even in reaction mixtures that contained elevated levels of CO_2_ that normally abolish the inhibitory effects of oxygen for form I and form II RubisCOs. The rather unique interactions of these archaeal RubisCOs with oxygen underscores the fact that, in general, the archaeal enzymes are less understood and characterized compared to their form I and form II counterparts. In addition, there are few substrate specificity values available in the literature for the archaeal form III proteins and these values appear to be widely discrepant for homologous proteins. For example, extremely high substrate specificity values; e.g., of 290 at 80 C and 310 at 90 C were originally reported for the enzyme from the hyperthermophile *Thermococcus kodakaraensis* [20], yet the enzyme from another hyperthermophile, *Archaeoglobus fulgidus*, which is 72% identical at the amino sequence level to the *T*. *kodakaraensis* protein, was recently reported to have an Ω value of 4 at 83 C [17]. The low specificity value for the *A*. *fulgidus* enzyme is in line with other values reported for archaeal RubisCOs [16–19]. Interestingly, two separate recent studies on the *T*. *kodakaraensis* enzyme from the same laboratory report specificity values of 11 [22] and 6 [21] at ambient temperature.

**Fig 1:**
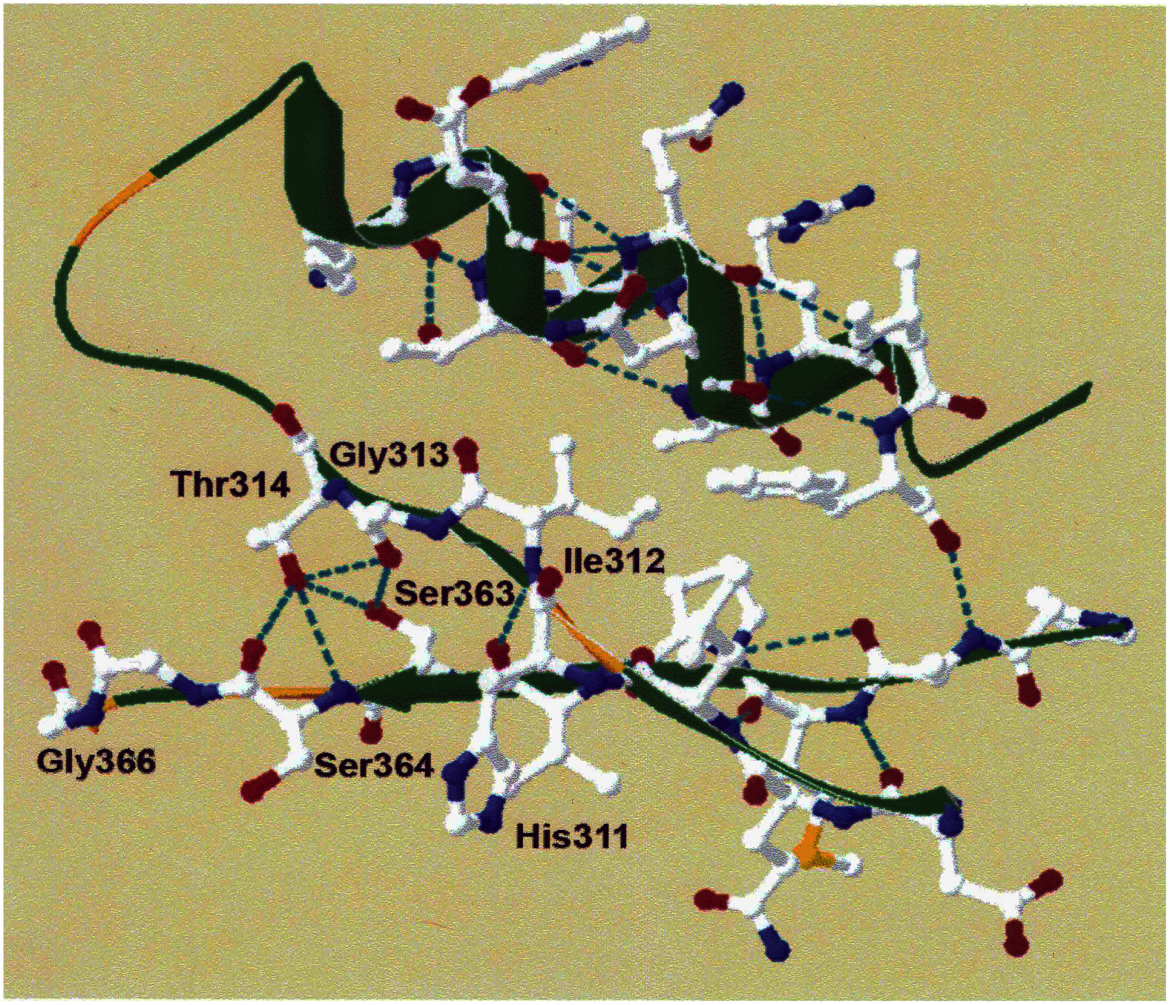
The hydrophobic pocket of *Archaeoglobus fulgidus* RbcL2 RubisCO showing interactions of Se∇r-363 with conserved residues Gly-313 and Thr-314. Ile-312 is situated on *β*–strand 6 between a highly conserved residue, His-311, necessary for the binding of RuBP and the Gly-313 residue that interacts with Ser-363. Ser-363 is directed away from the active site towards *α*–helix 6. The loop 6 structure is important by virtue of folding over the active site during catalysis and is between *β*–strand 6 and *α*–helix 6, shown on the upper left of the figure.

To gain a fuller understanding of the properties of the unusual RubisCOs from archaea, we have undertaken detailed studies of the *A*. *fulgidus* enzyme (RbcL2) [17]. Since such studies might shed light on the means by which RubisCO in general interacts with O_2_, we have particularly focused on probing the molecular basis for the interesting oxygen sensitivity exhibited by this enzyme. The facile production of recombinant protein suggested that a mutagenesis approach, combined with structural and bioinformatic insights, might be useful for such studies [17]. Initial analyses had shown that Met-295, when changed to a more polar, positively charged residue(aspartic acid), resulted in a protein with a significant alteration in its sensitivity to oxygen compared to the wild-type form of the enzyme. A nearly five-fold increase in the K_o_ for oxygen (24 *μ*M) for the M295D RbcL2 enzyme was obtained compared to the wild-type enzyme [17]. Moreover, studies of the *A*. *fulgidus* RbcL2 model structure compared to the structures of form I and II RubisCOs indicated the presence of unique interactions with known active site residues; in particular Ser-363 appeared to be a residue whose position was unique to form III proteins (Fig 1).

To assess the importance of Ser-363, in the current study mutations were made in archaeal RubisCO at this residue. Ser-363 resides in a hydrophobic pocket in which many of the constituent hydrophobic residues are conserved among form I, II and III RubisCOs [4–6, 21] (Table 1). However, only in representative form III archaeal RubisCOs is there a serine positioned in the pocket; the equivalent residue found in form I and II enzymes are hydrophobic. Substitutions were thus made so as to reflect the equivalent residues in form I (Ala) and form II (Ile) enzymes. Previously, it was shown that a Val substitution at the equivalent position (Ala-375) in the form I *Synechococcus* PCC6301 enzyme resulted in a mutant enzyme that was less inhibited by oxygen [23]. Thus, a Val substitution was also introduced into the *A*. *fulgidus* RbcL2 protein. Finally, to test whether the effects on oxygen insensitivity observed for *A*. *fulgidus* RbcL2 might also hold with another closely related form III archaeal RubisCO, similar alterations were made at equivalent sites in the homologous *T*. *kodakaraensis* RbcL. The results shown here describe the importance of a localized region, the hydrophobic pocket surrounding Ser-363, and how it may influence the enzyme’s CO_2_ and O_2_ substrate specificity.

**Table 1:** Amino acid residues involved in the formation of a hydrophobic pocket in a specific region of the RubisCO enzyme and sequence comparison of these residues between forms I, II and III RubisCOs.

*Syn*. PCC6301 RbcL (form I)	*R*. *rubrum* CbbM (form II)	*A*. *fulgidus* RbcL2 (form III)
Gly-326	Gly-323	Gly-313
Thr-327	Thr-324	Thr-314
Lys-331^a^	Lys-329^a^	Lys-319^a^
Leu-332	Leu-330	Leu-320
Val-373	Ile-365	Val-361
Pro-374	Pro-366	Pro-362
Ala-375	Ile-367	Ser-363
Ser-376 ^a^	Ser-368 ^a^	Ser-364 ^a^
Gly-378 ^a^	Gly-370 ^a^	Gly-366 ^a^
Ile-379	Met-371	Leu-367
Phe-391	Leu-383	Leu379
Gly-392	Gly-384	Gly-380
Val-396	Ile-390	Val-384
Leu-397	Leu-391	Ile-385

^a^ Catalytic residue

## Materials and Methods

### Plasmids, bacterial strains and growth conditions

Plasmids and bacterial strains used are summarized (S1 Table). *In vitro* studies of *A*. *fulgidus* RbcL2 and *T*. *kodakaraensis* RbcL were performed with recombinant protein prepared from *Escherichia coli*. All cloning steps were performed in *E*. *coli* JM109 [24] prior to transformation into *E*. *coli* BL-21(DE3) (Stratagene, La Jolla, California) for overexpression of the wild-type and mutant *A*. *fulgidus rbcL2* genes. *E*. *coli* cultures were grown in Luria-Bertani (LB) media containing 1% tryptone, 0.5% yeast extract, and 1% NaCl (w/v). *T*. *kodakaraensis rbcL* (Tk 2290, accession number NC_006624), was cloned directly from genomic DNA. Primers designed with an *Nde*I (5'GCATATGATGGTTGAGAAGTTTGATACGATATACGACTACTATGTTGACAAGGGCTACG3') restriction site at the N terminus and a *Bam*HI (5'GCGGATCCTCAGACTGGAGTAACGTGACCCCACTTCTCCAGGG3') restriction site at the C terminus were used to amplify the *rbcL* gene from *T*. *kodakaraensis* genomic DNA using Pfu polymerase. The gene was ligated into pCR2.1-TOPO vector (Invitrogen) and sequenced to determine if there were any PCR-incorporated mutations. Using the *Nde*I and *Bam*HI sites in that vector, the gene was subcloned into pET11a (Novagen). *A*. *fulgidus rbcL2* (Af 1638, accession number NC_000917), was cloned directly from genomic DNA as previously described [17].

*In vivo* studies of wild-type and mutant *A*. *fulgidus* RbcL2 were performed after complementation of the genes into the RubisCO deletion strain *Rhodobacter capsulatus* strain SBI/II^-^. Construction of *R*. *capsulatus* SBI/II^-^from wild-type strain SB1003 has been described [25]. All growth on plates and in liquid media for *R*. *capsulatus* was at 30°C. *R*. *capsulatus* was grown aerobically on peptone yeast extract (PYE) plates or in a liquid SOC media containing Ormerod’s basal salts as previously described and both were supplemented with 1 mg/ml of nicotinic acid and 1 mg/ml of thiamine-hydrochloride [25]. Antibiotics in PYE plates or in SOC media were used at the following concentrations: 100 μg/ml of rifampicin, 2 μg/ml of tetracycline, 10 μg/ml of spectinomycin, and 5 μg/ml of kanamycin. DL-malate was added to 0.4% (w/v) to basal salts for photoheterotrophic growth on plates and in liquid media. Minimal medium plates for photoautotrophic CO_2_-dependent growth, and minimal malate medium plates (0.4% malate) for photoheterotrophic growth, were incubated in jars containing a CO_2_/H_2_-generating system (5–6% CO_2_, BBL GasPak system, Becton Dickson Microbiology Systems, Cockeysville, MD). In some cases, photoautotrophic plates were grown under conditions where jars were flushed for 15 min with premixed 20% CO_2_/80% H_2_. All phototrophic jars contained a palladium catalyst to remove O_2_ from the atmosphere, and all jars were incubated in water-baths in front of lights. Growth curves for *R*. *capsulatus* SBI/II^-^ liquid cultures were generated by obtaining absorbance readings at 660 nm at intervals of 6 to 12 h. Minimal liquid medium for photoautotrophic growth and minimal malate medium for photoheterotrophic growth were set up in the anaerobic chamber in 25 ml sealed tubes containing 10 ml media. The tubes were capped with rubber stoppers and crimped inside the chamber. For photoautotrophic growth, the headspace was exchanged by removing the gas phase and sparging with premixed 20% CO_2_/80% H_2_ at 1 min intervals for three cycles, performed every 24 h. When cultures reached an A_660_ between 1.2 and 1.5, a turbidity range known to yield maximum RubisCO specific activity, the cells were harvested by centrifugation and then washed with 100 mM Bicine—NaOH (pH 8.3), 10 mM MgCl_2_, and the resultant cell pellets stored at -80°C.

Plasmid pRPS-MCS3 was constructed specifically for the complementation system, as previously described [23]. A pET11a clone of *rbcL2* from *A*. *fulgidus* that would facilitate directional cloning into pRPS-MCS3 was constructed by amplification of *rbcL2*. Primers designed with a *Kpn*I (5'CGGGTACCGTTGAAGATAAAACTTCTATCCCCC3') restriction site at the N terminus and a *Sac*I (5'GCGGAGCTCTTAGATTGGCGTAACCCTGCCC3') restriction site at the C terminus were used to amplify the *rbcL2* gene from the pET11a plasmid containing the gene using Taq polymerase. The gene was ligated into pRPS-MCS3 and sequenced to ensure the absence of PCR-incorporated mutations. The ligated plasmid was transformed in the *E*. *coli* JM109 host strain. *A*. *fulgidus rbcL2* wild-type and mutant genes were directionally cloned into the plasmid using blue-white screening to facilitate isolation of desired insertions. The pRPS-MCS3 plasmid harboring *A*. *fulgidus rbcL2* wild-type or altered genes was mobilized into *R*. *capsulatus in trans* using a triparental mating procedure described previously [25]. Selection for the introduced plasmids was carried out on PYE agar plates containing rifampicin and tetracycline (PYE^rif-tet^). These plates were incubated for three days before transconjugates were streaked for individual colonies onto a replicate PYE^rif-tet^ plate.

### Site-directed mutagenesis

Site-directed mutagenesis was performed using the QuikChange site-directed mutagenesis kit from Stratagene [26]. Automated sequencing was performed to confirm the sequences of altered genes using a 3730 DNA Analyzer system (Applied Biosystems) at the OSU Plant-Microbe Genomics Facility. The altered genes were inserted into fresh pET11a plasmid after digestion with *Nde*I and *Bam*HI.

### Overexpression of the *A*. *fulgidus rbcL2* and *T*. *kodakaraensis rbcL* wild-type and variant genes in small scale cultures for initial analysis

*E*. *coli* BL-21(DE3) cells with transformed pET11a vector containing *A*. *fulgidus rbcL2* or *T*. *kodakaraensis rbcL* were grown to an OD_600_ of 0.4 using a 50 ml Erlenmeyer flask containing 25 ml LB medium at 37°C at 120 rpm to minimize aeration. The temperature of the medium containing the cultures was then raised to 42°C by placing the flasks in a water bath for 30 min to facilitate expression of *E*. *coli* heat shock (chaperone) proteins encoded by the *dnaJ*, *dnaK* and *groESL* genes. This served to increase the amount of soluble recombinant protein. The cultures were then allowed to cool to room temperature before inducing with 0.1 mM Isopropyl-ß-D-thiogalactopyranoside (IPTG); the cultures were then shaken at 120 rpm for 16 h at room temperature. Cells were harvested to remove LB media and then washed with anaerobic wash buffer, 100 mM *N*,*N*-bis(2-hydroxyethyl) glycine (Bicine), pH 8.3, 10 mM MgCl_2_, 1 mM EDTA, flushed with argon and stored in an anaerobic chamber. Cells were centrifuged again in anaerobic centrifuge bottles containing screw caps with rubber seals. Cell pellets were recovered in the chamber and were then stored at -70°C before further protein purification by column chromatography. When multiple enzyme variants with several residue alterations were analyzed, a protocol was developed for facile isolation of nearly homogeneous enzyme preparations suitable for kinetic analysis. Cells were resuspended in wash buffer and were disrupted using a Retsch MM200 cell mill. Resuspended samples were mixed with glass beads (0.10–0.25 μm) in a 1:1 (v/w) ratio and were placed in 2 ml anaerobic centrifuge tubes with screw caps containing rubber seals. The samples were then placed in the cell mill and allowed to process for 9 min at a frequency of 30 sec^-1^ at 4°C. The samples were then centrifuged at 13,100 x *g* for 10 min at 4°C in an Eppendorf 5414R bench top centrifuge. Further processing of the samples was performed for the thermostable enzymes from *A*. *fulgidus* and *T*. *kodakaraensis*. The supernatant was collected in the anaerobic chamber and placed in 2.7 ml gas-tight and crimped glass serum vials and then taken outside of the anaerobic chamber and heat treated in a 90°C water bath for 15 min to precipitate labile *E*. *coli* proteins. After the heat step, the samples were placed on ice for 30 min. The samples were then taken back into the chamber and placed into fresh centrifuge tubes with rubber seals and centrifuged at 16,100 x *g* under the same conditions as previously mentioned. The heat stable supernatant, containing highly purified enzyme suitable for kinetic analysis, was collected and used for further experiments.

#### Purification of homogeneous recombinant wild-type and single mutant *A*. *fulgidus* RbcL2 and *T*. *kodakaraensis* RbcL proteins

*E*. *coli* BL-21(DE3) cells with transformed pET11a vector containing *A*. *fulgidus rbcL2* or *T*. *kodakaraensis rbcL* were grown using 2.8 l broad bottom flasks containing 2 l of LB media to an OD_600_ of 0.4 at 37°C and shaken at 120 rpm to minimize aeration. Growth conditions were identical to the small scale growth as previously described.

All preparations and manipulations of cell material were performed in an anaerobic chamber. Prior to column chromatography, cells were resuspended in wash buffer supplemented with 10 mM phenylmethylsulfonyl fluoride (PMSF) and 50 *μ*g/ml deoxyribonuclease I (DNase I) and disrupted using a pressurized French pressure cell (at 110,000 kPa) flowing directly into a sealed anaerobic serum vial sparged with argon gas. The lysed cells were then centrifuged at 16,000 x *g* at 4°C for 20 min in screw cap centrifuge tubes with rubber sealed caps. The supernatant was decanted into a serum vial and placed in a 90°C water bath for 20 min and then allowed to cool on ice for 1 h. The heat stable extract was transferred to a fresh screw cap centrifuge tube with rubber sealed caps and centrifuged at 30,000 x *g* at 4°C for 30 min. Supernatant from either the thermostable *A*. *fulgidus* RbcL2 or *T*. *kodakaraensis* RbcL-containing extracts were syringe filtered using 0.22 *μ*m filters for further purification via column chromatography.

Column chromatography was performed in the anaerobic hood using a Bio-Rad BioLogic HR Workstation. Purifications were similar for *A*. *fulgidus* RbcL2 and *T*. *kodakaraensis* RbcL. For *A*. *fulgidus* RbcL2 and *T*. *kodakaraensis* RbcL, syringe filtered heat-stable extract was loaded onto a Q-Sepharose strong anion exchange column equilibrated with wash buffer supplemented with 50 mM NaHCO_3_ and 10 mM *β*-mercaptoethanol, pH 8.3 (Buffer A) exactly as previously described [17]. Samples were eluted using a gradient of 0–2 M NaCl in Buffer A; recombinant RubisCO enzyme typically eluted at ~0.4 M NaCl. Fractions were monitored for activity using a modified protocol of the standard RubisCO assay under anaerobic conditions [17]. Fractions with high levels of activity were pooled and concentrated with a Millipore 30,000 MWCO concentrator and loaded onto a 110 ml Superose-12 gel filtration column. Peak fractions were pooled and further purified based on hydrophobic interaction using a phenyl-sepharose column exactly as previously described [17]. Samples were eluted with decreasing salt concentrations starting with 2 M (NH_4_)_2_SO_4_. Both recombinant proteins were found to elute at ~0.4 M (NH_4_)_2_SO_4_. Fractions were pooled and concentrated with a 30,000 MWCO Millipore concentrator using a centrifuge and then loaded onto a 1 ml G-25 desalting column to remove any remaining (NH_4_)_2_SO_4_. Purified protein was stored in 20% glycerol at -70°C in anaerobically sealed serum vials.

### Radiometric RubisCO assays

Purified recombinant enzymes were assayed for activity under a strict anaerobic atmosphere unless otherwise noted. The previously described assay was used and modified to optimize carboxylase activity [17]. Buffers and substrates were bubbled with argon gas in sealed glass serum vials prior to use. In an anaerobic chamber, enzyme was prepared in glass serum vials in 100 mM Bicine-NaOH, pH 8.3, 10 mM MgCl_2_, 1 mM EDTA, and 0.4 M NaCl. The Bradford method was used to determine protein concentrations; BSA was used as the standard as described [17].

#### Kinetic measurements

Purified enzymes were used for all kinetic measurements of *k*_cat_, K_C_, K_O_, K_RuBP_, and Ω. The K_C_ was determined under strict anaerobic conditions using sealed vials as previously described with few modifications [17]. Dilutions of [^14^C]-NaHCO_3_ were prepared in 100 mM Bicine—NaOH buffer with 10 mM MgCl_2_. The pH of the buffer was usually around 8.3, and the exact pH was recorded for each assay. Results were plotted using Sigma Plot 2002 v8.0, deriving the K_C_ and K_O_ by fitting values to a hyperbolic curve and double reciprocal plot. The concentration of CO_2_ was derived using the pH and the Henderson—Hasselbach relationship. Solubility of CO_2_ at 83°C was calculated from published values to obtain an equation that was extrapolated to 83°C (17). After determining the average volume of the glass vials (2.2 ml), various concentrations of oxygen were introduced into the vials by removing a certain percent of the anaerobic headspace and replacing it with the same amount of oxygen from a sealed serum vial sparged with ultrapure oxygen. Incubations with oxygen were for a minimum of 10 min prior to RuBP addition. Often times, the enzyme in buffer was gassed while on ice and then the assay was performed sometime later with the same results. The percentage of oxygen introduced to the vial was then used to determine how much oxygen (in μM) was present in the vial and then the solubility of oxygen was determined using solubility charts available from Unisys^®^.

The K_RuBP_ was measured similarly to the K_C_, determined under strict anaerobic conditions in sealed serum vials at 83°C [17]. Specificity was measured under conditions of saturating O_2_ (1.23 mM) with 200 mM NaHCO_3_ in 100 mM Bicine-NaOH (pH 8.3), 10 mM MgCl_2_ as previously described (17). The concentration of CO_2_ was calculated from the Henderson—Hasselbach relationship, as described above for K_C_. Specificity reactions were initiated by addition of [1-^3^H] RuBP, and incubated at 83°C for 2 h. The reaction was halted by the addition of 200 mM NaBH_4_ and incubated at room temperature for 15 min. Excess NaBH_4_ was consumed by the addition of 400 mM glucose and incubated for an additional 15 min at room temperature [27]. Samples were diluted with distilled water and products formed were separated from the enzyme by centrifugation in a Millipore 10,000 MWCO concentrator. The samples were frozen at -70°C until further use. Reaction products were separated with a MonoQ resin using a Dionex DX500 chromatography system (Dionex Corporation, Sunnyvale, CA) and detected with an in-line scintillation counter (IN/US β-Ram, Tampa, FL), as described [27].

#### Molecular modeling of *A*. *fulgidus* RbcL2

Modeling of the *A*. *fulgidus* RbcL2 was performed using Deep View Swiss PDB Viewer, spdbv 3.7 [28,29]. The template used to model the dimer form of the enzyme was the *T*. *kodakaraensis* KOD1 crystal structure [30] (PDB, 1geh), which was reasonable as it possesses 72% amino acid sequence identity.

#### Western immunoblots using polyclonal antibodies to archaeal RubisCO proteins

Antiserum directed against purified *A*. *fulgidus* RubisCO was prepared in rabbits by Cocalico Biologicals, Inc. (Reamstown, PA) and Western immunoblots were used to test the specificity of the antiserum. Proteins resolved by sodium dodecyl sulfate polyacrylamide gel electrophoresis (SDS-PAGE) [31]) were transferred to polyvinylidene difluoride membranes (Immobilon-P; Millipore, Bedford, MA) according to directions supplied by the manufacturer using a BioRad Transblot semi-dry transfer cell (BioRad, Hercules, CA). Washes and incubations with antibodies were carried out as described using antibodies directed against the archaeal RubisCO that was used at a dilution of 1:3000 [16,17]. Immunoblots were developed with the Attophos detection reagent according to the manufacturer’s instructions (Amersham, Buckinghamshire, England) and visualized with a Molecular Dynamics Storm 840 imaging system (Molecular Dynamics, Sunnyvale, CA).

#### Circular dichroism (CD) measurements

Far-UV CD measurements were taken using an Aviv model 62A DS spectrometer, scanning from 190 to 260 nm at 1 nm intervals with a bandwidth of 1 nm and a 2 sec signaling average; three scans were averaged for each sample. Data were collected using 1 cm quartz cuvettes with screw caps containing rubber septas with the temperature controller set to 83°C. CD scan samples were prepared under strict anaerobic conditions in the anaerobic chamber and protein concentrations were ~1 mg/ml in 20 mM Tris—HCl buffer, pH 8.3 and were placed in a 1 cm path length quartz cuvette with screw cap lid and rubber septa. Samples were allowed to equilibrate at 83°C for 30 min. The cuvettes were then flushed with pure oxygen through the rubber septa for 30 min at room temperature and afterwards data was collected similar to the anaerobic samples at 83°C. Data was smoothed by applying a negative exponential 10 degree polynomial regression with SigmaPlot v8.0.

## Results

### Oxygen effects on *A*. *fulgidus* RubisCO variants (mutant enzymes)

**Fig 2:**
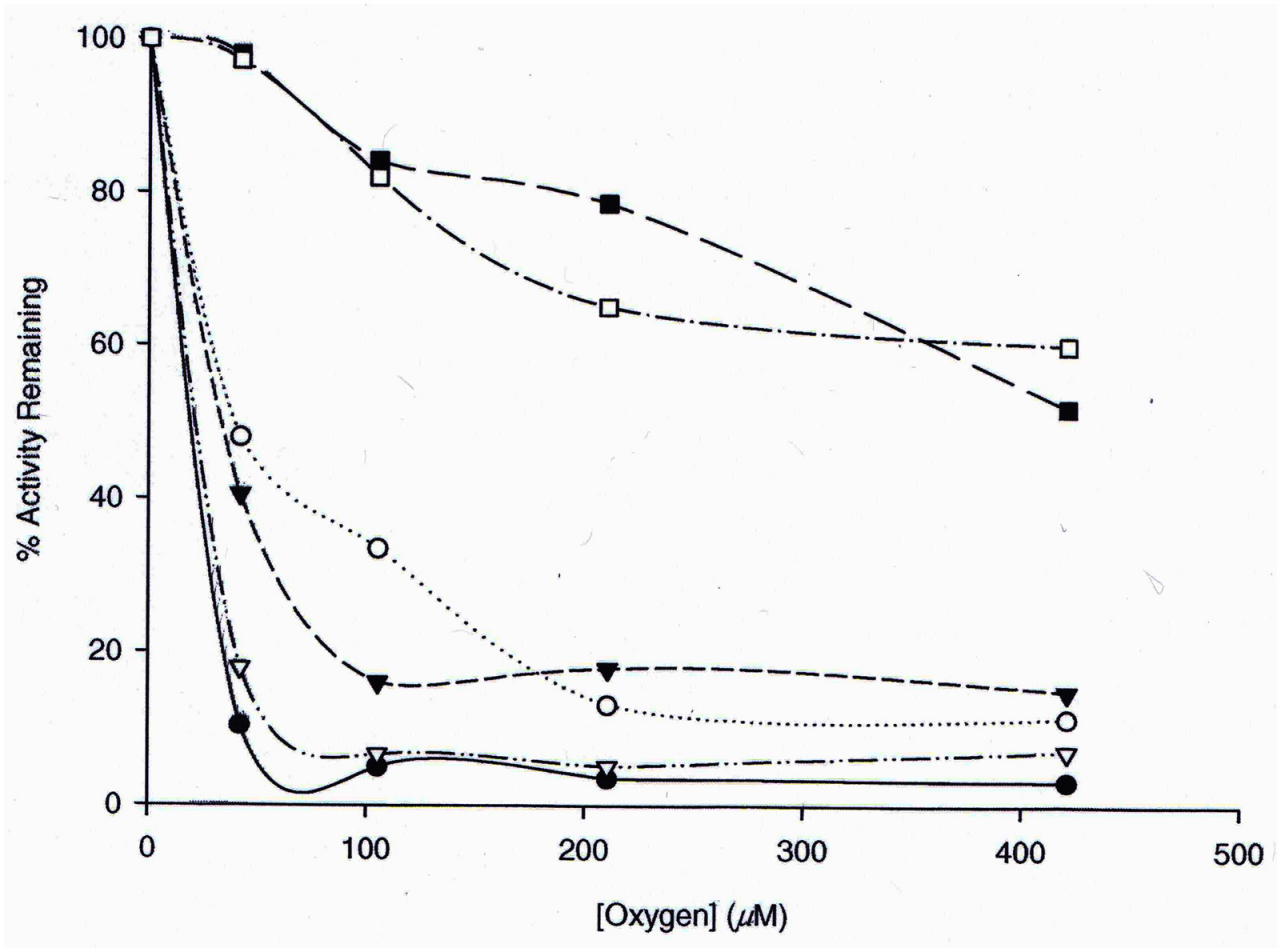
Retention of carboxylase activity in the presence of oxygen. Wild-type (●), M295D (○), S363I (▼), S363V (∇), M295D/S363I (■) and M295D/S363V (□) homogeneous enzymes were exposed to varying amounts of oxygen and assayed for carboxylase activity. As described in Materials and Methods, enzymes were exposed to oxygen for a minimum of 10 min, with no discernible change in activity after this time period. The percent activity retained is the difference in activity between the anaerobic samples compared to the oxygen exposed samples. The M295D/S363I and M295D/S363V enzymes retained significantly more activity than all the other enzymes when all enzymes were incubated with concentrations of oxygen ranging from 10% (42.1 μM) to 100% (421 μM) in the gas phase. Points represent the average of three determinations.

Previous studies focused on residues of *A*. *fulgidus* RbcL2 involved in the reversible inhibition by low concentrations of oxygen. Met-295 and Ser-363 appear to be located at two influential sites within the structure of the protein; these two residues may interact with catalytically important residues to alter kinetic parameters in the enzyme [17]. Ser-363, roughly 10 Ǻ from Met-295 according to the modeled structure, is situated in what appears to be a hydrophobic pocket that surrounds one side of the active site. The hydrophobic pocket appears to be highly conserved among RubisCO enzymes; residues that surround Ser-363, or its equivalent residue in other enzymes, are either identical or at least hydrophobic (Table 1). In addition, the *A*. *fulgidus* RbcL2 model structure shows an interaction of the side chain of Ser-363 with highly conserved and mechanistically significant/catalytically important residues Gly-313 and Thr-314 [11] (Fig 1). This unique interaction and positioning of Ser-363 in a key hydrophobic pocket of *A*. *fulgidus* RbcL2 thus suggested that Ser-363 of *A*. *fulgidus* RbcL2 might be a likely candidate for further investigation by site-directed mutagenesis. Previous studies had already implicated this residue, along with Met-295, for its role in influencing the enzyme’s response to oxygen exposure [17]. Moreover, Ser-363, or its equivalent residue in other organisms, abuts a conserved active-site Ser residue [e.g., Ser-364 of *A*. *fulgidus* RubisCO (Fig 1)] that is involved with binding RuBP [11] and had been previously shown to influence catalytic properties of the *R*. *rubrum* [32], cyanobacterial [33] and *Chlamydomonas* [34] RubisCOs, with the K_O_ substantially affected in the latter enzyme. To discern the differential effects of molecular oxygen, each of the purified Ser-363 mutant proteins described in this study, along with wild-type RbcL2, was exposed to increasing levels of molecular oxygen. The mutant enzymes retained significantly more activity than the wild-type enzyme when all enzymes were incubated with concentrations of oxygen ranging from 10% (42.1 μM) to 100% (421 μM) in the gas phase (Fig 2). Clearly, these enzymes were altered in such a way that the normal response to molecular oxygen was changed; the mutant enzymes, especially the M295D/S363I protein, appeared much less susceptible to the deleterious effects of oxygen.

#### Further characterization and determination of kinetic parameters of mutant *A*. *fulgidus* RbcL2 proteins

As with the wild-type and M295D enzymes [17], the kinetic constants for each of the substrates (K_C_, K_O_, and K_RuBP_ and Ω) were determined for the S363I, S363V, M295D/S363I and M295D/S363V enzymes at 83°C. The results showed that there was little change in the K_C_ for the S363I, S363V, M295D/S363I and M295D/S363V enzymes (Table 2). In agreement with the recovery experiment, there was an approximate 3-fold increase in the K_O_, from 5 μM for the wild-type enzyme to 18 μM or 16 μM determined for the S363I and S363V proteins, respectively. Surprisingly, there was a substantial increase in the K_O_ for the double mutants M295D/S363I and M295D/S363V with calculated values of 427 and 91 μM, respectively. These values are comparable to the K_O_ values for form I and II enzymes which are able to maintain carboxylase activity in the presence of oxygen [3] and these mutant enzymes showed the expected competitive inhibition by O_2_ with respect to CO_2_. In addition, the K_RuBP_ values determined for the S363I, S363V, M295D/S363I and M295D/S363V mutants were significantly higher than the wild-type enzyme (Table 2) in particular double mutants M295D/S363I and M295D/S363V, which have values of 1646 and 1381 μM, respectively. It is notable that the substrate specificity factor (Ω) for all the mutant enzymes increased relative to the wild-type protein, undoubtedly as a consequence of the increase in the K_O_/K_C_ ratio (Table 2).

**Table 2:** Kinetic properties of homogeneous recombinant wild-type and mutant RubisCOs from *A*. *fulgidus* RbcL2 and wild-type RubisCO from *T*. *kodakaraensis* RbcL assayed at 83°C. Kinetic constants were determined as previously described [17].

Enzymes	K_c_ ^a^	K_o_ ^a^	K_c_/K_o_	K_RuBP_ ^a^	*k*_cat_ ^b^	Ω ^a^(V_c_K_o_/V_o_K_c_)
	μM	μM		μM	s^-1^	
*A*.*ful*. RbcL2 Wt^c^	51 ± 8	5 ± 1	10.2	20 ± 5	23.1	4 ± 1
M295D^c^	58 ± 11	24 ± 7	2.4	21 ± 3	17.7	13 ± 1
S363I	79 ± 3	18 ± 2	4.3	570 ± 94	9.5	9 ± 2
S363V	88 ± 7	16 ± 1	5.5	118 ± 6	12.4	8 ± 1
M295D/S363I	74 ± 5	427 ± 96	0.2	1646 ± 310	0.7	9 ± 1
M295D/S363V	62 ± 17	91 ± 9	0.7	1381 ± 88	4.3	9 ± 0.7
*T*.*kod*. RbcL Wt	79 ± 5	43 ± 2	1.8	14 ± 2	16.6	6 ± 0.2

^a^ Average of at least three independent assays.

^b^ Determined at 20 x K_RuBP_ except for the S363V and M295D/S363I and M295D/S363V mutant enzymes which were determined at 4–6 x K_RuBP_.

^c^ Values for WT and M295D previously reported (17) and run in parallel with the other mutant *A*. *fulgidus* and *T*. *kodakaraensis* enzymes for comparison

#### Potential for detectable conformational changes

CD scans and discontinuous nondenaturing PAGE (13%) gels were utilized to test whether or not large conformational changes accompanied the observed activity changes and lower sensitivity to molecular oxygen, most notably in double mutants M295D/S363I and M295D/S363V. Data for the anaerobic samples was collected first (S1 Fig). Variation in the molar ellipticity between samples can be attributed to the deviation in protein concentration. Clearly, there were no significant discernible or detectable alterations to secondary structures, *α*-helix, *β*-sheets and loops in the mutant proteins (S1 Fig). The cuvettes were then flushed with pure oxygen through the rubber septa for 30 min at room temperature and afterwards data were collected similar to the anaerobic samples at 83°C. Anaerobic versus oxygen exposed samples were compared and similar results were obtained in all cases with all mutant and wild-type proteins (S2 Fig).

Finally, to supplement the CD results obtained with the wild-type and mutant *A*. *fulgidus* RbcL2 proteins, discontinuous nondenaturing PAGE (13%) gels were run under aerobic conditions on the lab bench as well as under anaerobic conditions in the anaerobic chamber. Results for aerobic nondenaturing PAGE gel electrophoresis were identical to gels performed anaerobically in the anaerobic chamber and were consistent with CD scans (S3 Fig).

#### Additional substitutions

To assess whether the specific properties shown by the M295D, S363I and S363V RbcL2 proteins are attributable to the uniqueness of these residue positions, substitutions were created at other positions in close proximity to these two residues The objective was to determine if a substitution close to these two residue positions could “fix” the kinetic properties of either the single or double mutant forms of the enzyme, such that the values would be reflective of the wild type. In the model structure of *A*. *fulgidus* RbcL2, Ile-312 resides on *β*-strand 6, most notably between His-311 and Gly-313 (Fig 1). Not only is His-311 catalytically important for the binding of RuBP during catalysis (11), but there are implications based on solved crystal structures of form I and II enzymes that the carbonyl group on the peptide backbone of this amino acid interacts and perhaps stabilizes a neighboring, highly conserved and catalytically important arginine residue (Arg-279 in *A*. *fulgidus* RbcL2). However, the model structure of *A*. *fulgidus* RbcL2 did not show such an interaction [17]. Based on these observations and the unique interactions observed in *A*. *fulgidus* RbcL2, Ile-312 was deemed to be a good candidate for further studies. Ile-312 is directed away from the active site and towards *α*-helix 6. In addition, there appears to be no interactions with surrounding residues. In form I and form II enzymes, the equivalent residue at this position is an alanine, serine or a threonine; thus a less bulky hydrophobic or a charged residue could have some impact in this localized region of the *A*. *fulgidus* enzyme (Fig 1). With these considerations in mind, Ile-312 was changed to alanine, serine and threonine to mimic the residues found at the same position in form I and II enzymes. Single mutations as well as a combination of double mutants and triple mutants with M295D, S363I or S363V were made. Since there were so many different constructs to analyze, we used partially purified preparations after a 90°C heat treatment of crude *E*. *coli* extracts as described in Materials and Methods. This treatment removed substantial amounts of extraneous protein and the end-result was highly active, yet not completely homogeneous, preparations of this thermophilic RubisCO. To monitor activity, each sample was assayed under strictly anaerobic conditions; then an aliquot of this enzyme preparation was exposed to molecular oxygen and re-assayed. Initial analyses of recombinant mutant enzymes I312A, I312S and I312T indicated that they retained 30%, 16% and 15% activity when exposed to oxygen compared to the enzyme assayed under anaerobic conditions (S2 Table). The double mutants, M295D/I312A., M295D/I312S and M295D/I312T showed roughly the same response to oxygen sensitivity, 40%, 46% and 45%, respectively, of the anaerobically assayed enzyme, much like the previously studied single mutant M295D. Also, much like the absolute activity levels (specific activities or *k*_cat_) of the M295D mutant being similar or higher than the wild-type enzyme, the two double mutations also had the same levels of activity as the wild-type enzyme, unlike the low levels of activity observed with double mutant enzymes M295D/S363I and M295D/S363V (S2 Table) [17]. Double mutations of I312A/S363I and I312S/S363I were constructed and the oxygen sensitivity results (75 and 78% activity remaining, respectively, after exposure of anaerobic preparations to oxygen) were similar to that obtained with a single mutation to Ser-363, but not to the single Ile-312 mutant enzyme. This level of recovery after oxygen exposure was a bit less than what was routinely obtained with the other double mutant enzymes, M295D/S363I and M295D/S363V. Clearly, the double mutations of I312 with either M295 or S363 do not impose as much of a significant effect as compared to M295D/S363 double mutations.

To further probe the effect of changes in residues Met-295, Ile-312 and Ser-363, triple mutant-substitutions were constructed and analyzed. When assayed anaerobically, the M295D/I312A/S363I, M295D/I312S/S363I, M295D/I312A/S363V and M295D/I312S/S363V enzymes all had substantially lower levels of activity in comparison to the single as well as double mutant- proteins, even when assays were performed in the presence of extremely high concentrations of RuBP to account for the possibility that the enzymes may have very high K_RuBP_ values. The specific activities of the M295D/I312A/S363I and M295D/I312S/S363I enzymes were particularly low, i.e., 51 and 46 nmol/min/mg, respectively, while the specific activities of the M295D/I312A/S363V and M295D/I312S/S363V enzymes was somewhat higher, 146 and 140 nmol/min/mg, respectively.

In summary, it appears that as a consequence of changing these three residues near the active site, absolute activity levels (specific activities or *k*_cat_) of single-mutant proteins were similar or slightly lower than the values for the wild-type enzyme; double-mutant proteins, with the exception of M295D/I312A. M295D/I312S, M295D/I312T, had substantially lower levels of activity and the activity levels for the triple mutant proteins were significantly lower than the values for the wild type, single and double-mutant enzymes (S2 Table).

### Complementation of bacterial RubisCO deletion strain with the *A*. *fulgidus rbcL2* genes

**Fig 3:**
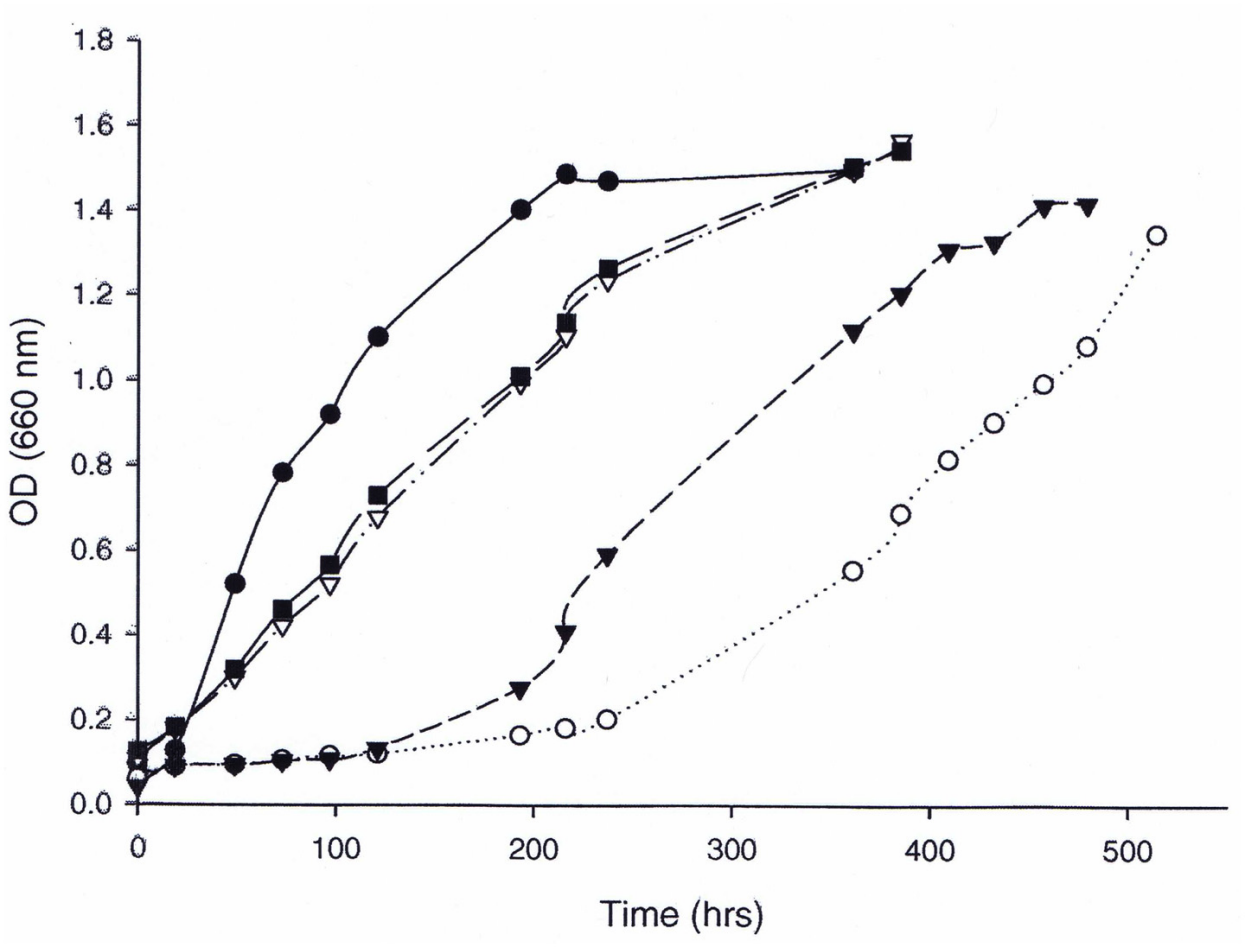
Complementation and growth of *R*. *capsulatus cbbLS*/*cbbM* knockout strain SBI/II^-^ using *A*. *fulgidus* wild-type and mutant Rubisco (*rbcL2*) genes in plasmid pRPS-MCS3MA. Photoautotrophic growth was performed under an atmosphere of 20% CO_2_/80% H_2_ in glass tubes prepared under anaerobic conditions. Wild-type *R*. *capsulatus* SB1003 (●); *R*. *capsulatus* strain SBI-II- containing plasmid pRPS-MCS3 and *A*. *fulgidus rbcL2* wild type (○) or mutants M295D (▼); S363I (■); and S363V (▽). Points represent the average of two to three cultures.

The RubisCO deletion strain of *Rhodobacter capsulatus* (strain SBI/II^-^) cannot grow under phototrophic conditions. Previous studies in this laboratory indicated that *in trans* introduction of form III mesophilic archaeal RubisCO genes allowed phototrophic growth of strain SBI/II^-^ [16]. In particular, *Methanosarcina acetivorans rbcL* [35,36] was able to complement growth under photoheterotrophic and photoautotrophic conditions so long as strict measures were taken to insure anaerobicity [16]. Analogous growth complementation studies with the form III archaeal RubisCO gene from *T*. *kodakaraensis* was recently accomplished using a strain of *Rhodopseudomonas palustris* in which the three endogenous sets of RubisCO genes were knocked out [22]. Since *A*. *fulgidus* RbcL2 has a temperature range of activity from 23°C to 93°C, much like its homolog *T*. *kodakaraensis* RubisCO, attempts were undertaken to complement *R*. *capsulatus* SBI/II^-^ with the *A*. *fulgidus rbcL2* gene at 30^°^C. Such studies with wild-type and altered enzymes provide a physiological context to any changes that were examined in vitro. Photoheterotrophic (malate minimal medium) growth tubes used in these experiments were allowed to equilibrate in the anaerobic chamber; in addition all inoculations into the culture tubes were performed in a strictly anaerobic environment. Under such conditions, it was found that the *A*. *fulgidus rbcL2* gene was expressed in *R*. *capsulatus* strain SB I/II^-^ and that this recombinant archaeal enzyme could complement growth under anaerobic photoheterotrophic conditions. In addition, SDS-PAGE and Western immunoblot experiments provided further evidence that the *A*. *fulgidus* gene was expressed to low levels (as expected) in the *R*. *capsulatus* RubisCO deletion strain under photoheterotrophic growth conditions (S4 Fig). Radiometric RubisCO assays performed on crude extracts from photoheterotrophically grown cells further indicated that RubisCO activity levels were low under these conditions and was strictly dependent on anaerobiosis. The levels of activity were substantially enhanced when assays were performed at 83°C, in keeping with the observed properties of the *A*. *fulgidus* enzyme (S3 Table). Since the CBB pathway and a functional RubisCO are absolutely required when these organisms are grown photoautotrophically under a H_2_/CO_2_ environment [25,34], It was determined whether *A*. *fulgidus rbcL2* expression would support photoautotrophic (CO_2_-dependent) growth of the *R*. *capsulatus* RubisCO knockout strain. Again, complementation to photoautotrophic growth depended on strict anaerobiosis. Sealed and crimped tubes prepared in the anaerobic chamber were used in these experiments and it was seen that *A*. *fulgidus* RbcL2 RubisCO constructs supported growth in the *R*. *capsulatus* RubisCO knockout strain under a gas phase of 20% CO_2_ balanced with hydrogen (Fig 3). Indeed, because it is difficult to exclude oxygen completely from the growth tube apparatus, archaeal RubisCO and CO_2_-dependent growth of *R*. *capsulatus* strain SBI/II^-^ often proceeds with a lag, e.g., until the organism can remove any vestiges of oxygen in the system [16]. Interestingly, it appears that mutant enzymes that are less oxygen sensitive compared to the wild-type RbcL2 enzyme supported growth with considerably shorter lag times compared to the wild-type enzyme (Fig 3). SDS-PAGE and Western immunoblot experiments provided further evidence that the *A*. *fulgidus* gene was highly expressed in the *R*. *capsulatus* RubisCO deletion strain under photoautotrophic growth conditions (S5 Fig) and radiometric assays indicated that RubisCO activity was greatly enhanced at 83°C when cells were grown photoautotrophically (S3 Table). After both photoheterotrophic and photoautotrophic growth of the complemented strains, plasmid pRPS-MCS3 (containing either the wild-type or mutant *A*. *fulgidus rbcL2* genes) was re-isolated from *R*. *capsulatus* SBI/II^-^ and sequenced to determine if there were any point mutations that may have been selected under these growth conditions. No additional mutations were detected. Subsequent reintroduction of the plasmid into a new SBI/II^-^ background again resulted in photoheterotrophic and photoautotrophic growth, eliminating the possibility that mutations in strain SBI/II^-^ somehow allowed complementation.

### Oxygen interactions with the form III *T*. *kodakaraensis* RubisCO

**Fig 4:**
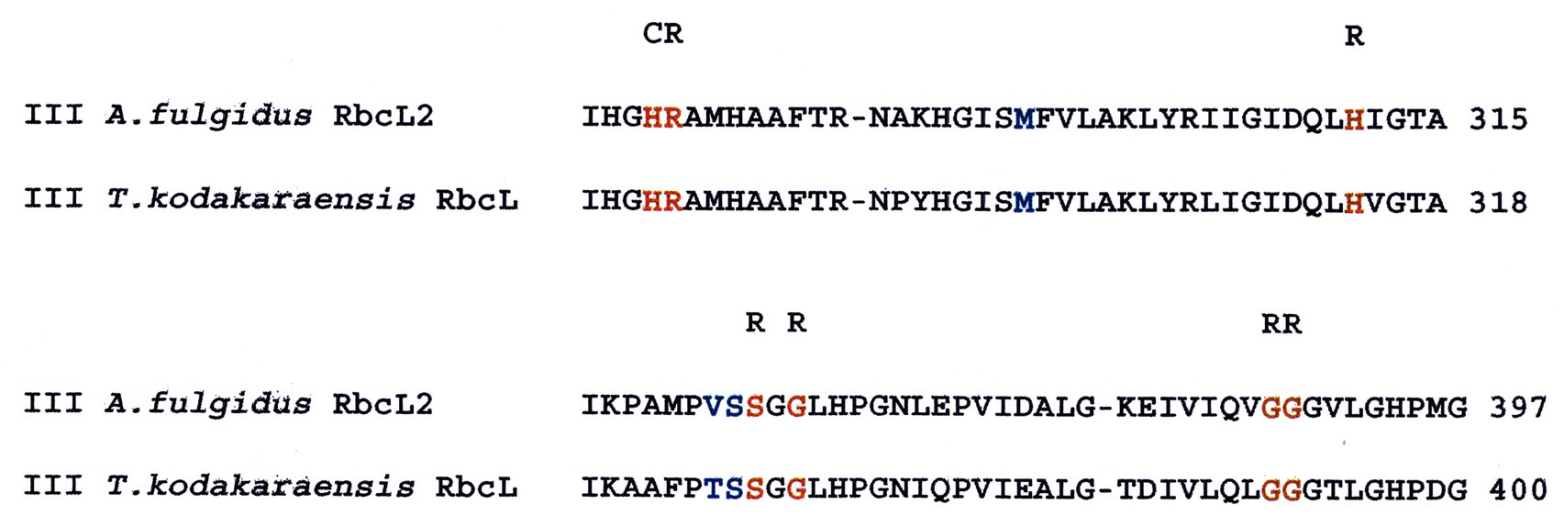
Partial amino acid sequence alignment of *A*. *fulgidus* and *T*. *kodakaraensis* archaeal form III RubisCOs. Multiple sequence alignments were performed by using ClustalW [39]. Residue identities are marked with an asterisk, conserved substitutions are marked with a colon, and semiconserved substitutions are marked with a period. Known active-site and highly conserved residues are labeled C for catalytic and R for RuBP binding properties and colored red. Amino acids colored blue are identical to the position of either Met-295 or Ser-363 residues in the model structure of *A*. *fulgidus* RbcL2.

**Fig 5:**
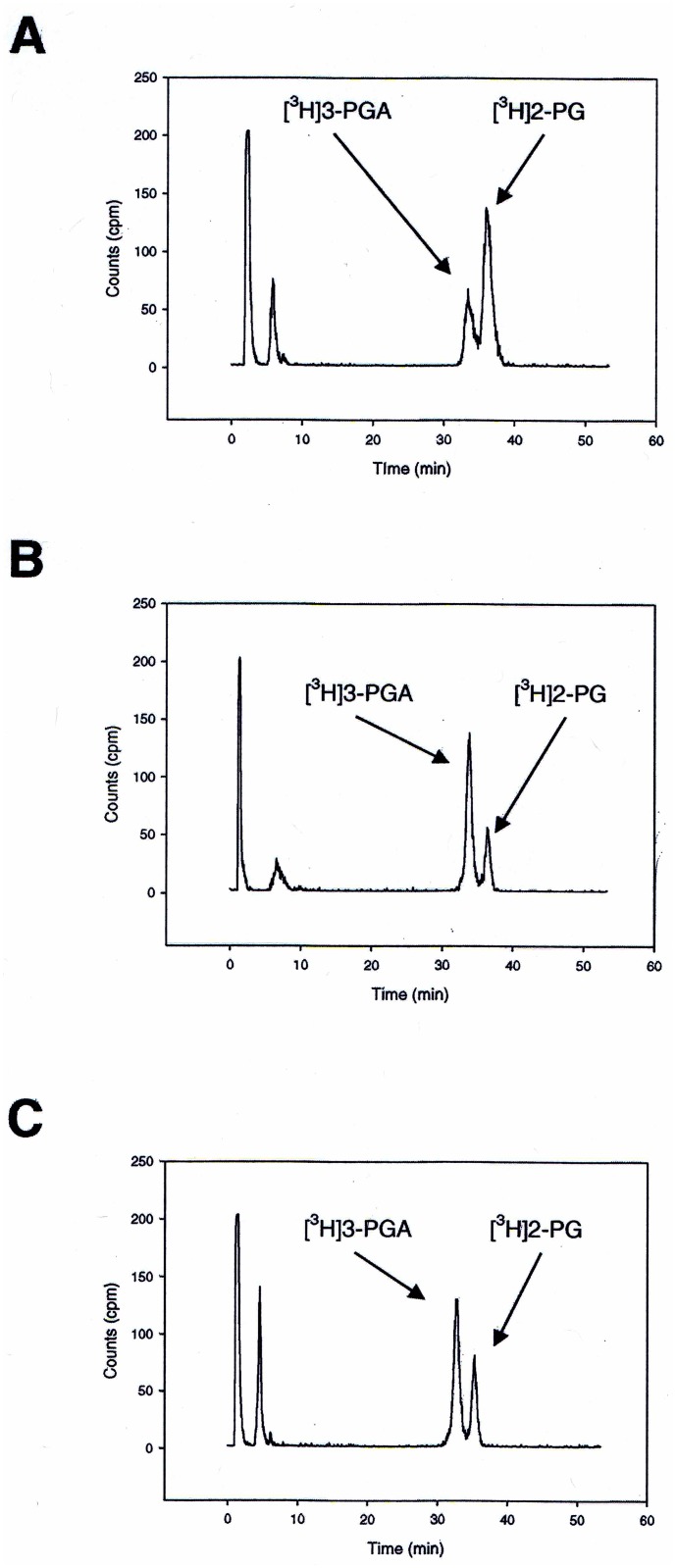
Anion exchange chromatographic separation of RubisCO reaction products [^3^H]3-PGA and [^3^H]2-PG generated from a completed reaction mixture containing [1-^3^H] RuBP after 2 h reaction at 83°C. *T*. *kodakaraensis* RubisCO was incubated in the presence of both molecular oxygen and CO_2_ to generate [^3^H] 3-PGA and [^3^H] 2-PG under (A) defined conditions as described in Materials and Methods and (B) under conditions previously described [20]. In (C), wild-type *A*. *fulgidus* RbcL2 was assayed under the same conditions as previously described [20]. Peaks at the beginnings of the chromatographic profiles represent degraded RuBP produced in this reaction mixture at high temperatures.

It was of interest to determine if residues that affect the response to molecular oxygen for the *A*. *fulgidus* RbcL2 play a similar role in other archaeal RubisCOs. *T*. *kodakaraensis* RbcL is a form III archaeal RubisCO that is a homolog to *A*. *fulgidus* RbcL2 (72% sequence identity) but was initially described as not being inhibited by oxygen [18]. Moreover, the crystal structure of the *T*. *kodakaraensis* RbcL has been solved [21,37,38] and of course this protein has served as the template for the molecular modeling of *A*. *fulgidus* RbcL2. When aligned, Met-298 and Ser-366 in *T*. *kodakaraensis* RbcL correspond to Met-295 and Ser-363, respectively, of *A*. *fulgidus* RbcL2 (Fig 4). Using site-directed mutagenesis protocols, Met-298 was changed to an aspartic acid residue and Ser-366 was changed to either an isoleucine or valine residue. The genes corresponding to wild type, single-mutants M298D, S366I and S366V, and the double mutants, M298D/S366I and M298D/S366V were all expressed in *E*. *coli* BL-21(DE3) and recombinant proteins were prepared from small-scale growth cultures, as described in Materials and Methods. Since *T*. *kodakaraensis* is also a hyperthermophilic organism the RbcL enzyme had the same basic heat stability as *A*. *fulgidus* RbcL2. Thus, the enzyme was highly purified after simply heating crude extracts to 90°C for 15 min followed by immersion into an ice bucket and subsequent removal of denatured protein by centrifugation. Assays indicated that such preparations of wild-type *T*. *kodakaraensis* RbcL possessed high specific activity at high temperatures (83°C) under strict anaerobic conditions similar to what was observed with wild-type *A*. *fulgidus* RbcL2. Likewise, after exposure to oxygen, the activity of the *T*. *kodakaraensis* RbcL decreased by 37% when compared with enzyme preparations that were kept anaerobic (Table 3). This loss of activity upon oxygen exposure was not nearly as severe as that obtained with the wild-type *A*. *fulgidus* RbcL2 enzyme (10–15% activity remaining) under the same conditions [17]. These initial results prompted further studies on the effects of molecular oxygen with purified wild-type and mutant *T*. *kodakaraensis* RbcL under our conditions of assay. Thus, over-expression of the various *T*. *kodakaraensis rbcL* genes was performed with large scale cultures and the resultant heat stable recombinant RbcL proteins were purified to homogeneity by column chromatography as described in Materials and Methods. These preparations were examined by SDS and nondenaturing PAGE and the results indicated that the enzyme was of high purity and most contaminating proteins had been removed (S6 Fig). The native PAGE gel indicated that the *T*. *kodakaraensis* RbcL, which is reported to be a decamer [28,36], migrated much slower than the *A*. *fulgidus* enzyme, which is a dimer (17) (S7 Fig). Under strictly anaerobic conditions, with assays performed at 83°C, the purified wild-type *T*. *kodakaraensis* RbcL enzyme had a high specific activity of 20 *μ*mol/min/mg. After exposure to molecular oxygen via the usual protocols, the specific activity of the purified enzyme was 49% of that compared to enzyme maintained under anaerobic conditions, indicating that the *T*. *kodakaraensis* RbcL is clearly oxygen sensitive. In part these studies were undertaken because of the rather surprising CO_2_/O_2_ substrate specificity values of 290–310 at 80 to 90°C previously reported [20]. Such results would indicate an extraordinarily highly favorable carboxylase reaction that is not inhibited by the presence of oxygen. Using precisely defined conditions (Materials and Methods) CO_2_/O_2_ specificity (Ω) values of the purified wild-type *T*. *kodakaraensis* RbcL enzyme were determined. The Ω was found to be 6 ± 0.2 (Table 2, Fig 5A). Attempts to assay the enzyme under the high CO_2_ concentration conditions described by Ezaki *et al*. [20] were performed at 83°C; however the correct specificity value (Ω) could not be accurately calculated because these authors did not specify the precise concentrations of gaseous CO_2_ and O_2_ substrate concentrations used in their assays. Thus, even though the resulting chromatogram displays formation of the characteristic separated product peaks (3-phosphoglyceric acid and 2-phosphoglycolate), and the expectedly large 3-phosphoglyceric acid peak (Fig 5B and 5C), it was not possible to accurately calculate the CO_2_/O_2_ specificity (Ω) value for both *T*. *kodakaraensis* RbcL and *A*. *fulgidus* RbcL2 under these conditions, simply because the concentrations of the gaseous substrates were not specified by these authors. Since the specificity value (Ω) for the wild-type *T*. *kodakaraensis* RbcL RubisCO at 83°C could be determined under our highly defined conditions, it was desirable to determine the key kinetic constants for each of the substrates (K_C_, K_O_, and K_RuBP_). The K_C_ value was determined to be 79 ± 5 *μ*M (Table 2). Similar to kinetic assays performed for the determination of K_O_ for *A*. *fulgidus* RbcL2 [15], the K_O_ was determined to be 43 ± 2 *μ*M (Table 2), a value that is considerably higher than the *A*. *fulgidus* protein, perhaps reflective of the fact that the *T*. *kodakaraensis* protein is less oxygen sensitive than it’s *A*. *fulgidus* homolog. Unlike previous experiments with this enzyme [20], these results clearly demonstrate that many of the basic kinetic properties of *T*. *kodakaraensis* RbcL are very similar to those recently determined for *A*. *fulgidus* RbcL2. In retrospect, this does not appear to be unusual due to the fact that these two enzymes are so closely related. Moreover, previously claimed specificity values of 310 at 90°C [20], would indicate that this enzyme would be essentially insensitive to oxygen and would represent an enzyme that possessed virtually only carboxylase activity with the highest reported specificity value for any RubisCO at its temperature optimum. The data reported here do not support this assertion.

**Table 3:** Carboxylase activity at 83°C of highly purified recombinant *T*. *kodakaraensis* RbcL wild-type and mutant enzymes under anaerobic and oxygen exposed conditions.

Enzymes	Anaerobic Carboxylase Activity (μmol/min/mg)^a^	O_2_ Exposed Carboxylase Activity (μmol/min/mg)^a^	% Activity Retained
Wild-type	6.13	2.33	37
M298D	4.90	2.35	51
S366I	0.83	0.60	75
S363V	4.18	2.85	71
M295D/S363I	0.004	0.003	75
M295D/S363V	0.028	0.021	75

^a^ Average of duplicate assays

#### The effect of mutations in *T*. *kodakaraensis* RbcL

Substitutions were made at positions Met-298 and Ser-366 in *T*. *kodakaraensis* RbcL. The residue changes were equivalent to the single and double substitutions made in *A*. *fulgidus* RbcL2. Heat stable extracts were prepared from *E*. *coli* cultures as described above and these highly purified samples were assayed at 83°C under strict anaerobic conditions as well as after exposure to molecular oxygen. The results were similar to what had previously been obtained for heat stable highly purified extract preparations of wild-type *A*. *fulgidus* RbcL2. The M298D, S366I and S366V *T*. *kodakaraensis* enzyme variants had slightly lower activities compared to wild-type RbcL, however these samples retained higher activities after oxygen exposure, averaging 51, 75, and 71%, respectively, compared to the wild-type enzyme (Table 3). The double mutants of M298D/S366I and M298D/S366V lost a significant amount of activity compared to the wild-type enzyme, however they still maintained high levels of activity after oxygen exposure, 78 and 75%, respectively (Table 3).

## Discussion

### *A*. *fulgidus* RbcL interactions with oxygen

In previous studies, it was demonstrated that purified *A*. *fulgidus* RbcL2 RubisCO is functional under strict anaerobic conditions at high temperatures and has substantial, but reversible, sensitivity to oxygen [17]. Since *A*. *fulgidus* is a thermophilic obligate anaerobe isolated from the bottom of the ocean near hydrothermal vents, it is not surprising that RubisCO from this organism is adapted to function under similar extreme conditions in vitro. Thus far, it appears that this response to oxygen has been observed only for form III archaeal RubisCOs, including the enzymes from both mesophilic and thermophilic archaea such as *M*. *acetivorans*, *M*. *burtonii*, *M*. *jannaschii*, *A*. *fulgidus* and *T*. *kodakaraensis* [16,17,19, this study]. Coupled with the solved crystal structure of the related RubisCO from *T*. *kodakaraensis* [21,30,37,38], it was deemed feasible to continue investigating the molecular basis for the unique interactions with molecular oxygen exhibited by the *A*. *fulgidus* enzyme [17].

In previous studies, the effect of a methionine to aspartate substitution at position 295 was analyzed [17]. In addition to Met-295, analysis of the linear sequence of RbcL2 from *A*. *fulgidus* and other archaeal RubisCOs compared to other well-studied form I and form II enzymes brought attention to another residue, Ser-363. Using homology modeling, Ser-363 was found to be in close proximity to the active site; this loop structure positioned in a distinct hydrophobic pocket, as illustrated in Fig 1. Many of the amino acids that surround this serine residue are highly conserved in form I, II and III RubisCOs (Table 1). After altering this residue by site-directed mutagenesis and preparing recombinant S363I and S363V proteins, it was apparent that these enzymes showed substantially less sensitivity to molecular oxygen than the wild-type protein, much like the M295D mutant protein [17] (S2 Table). Thus, Ser-363 should play an important role in the enzyme’s ability to retain activity in the presence of oxygen, similar to M295D. This is supported by previous findings of increased activity retention when enzymes were assayed in the presence of oxygen. Perhaps this effect is caused by either a disruption of the Van der Waals interactions between Gly-313, Thr-314 and Ser-363 or due to localized structural changes in the area of this hydrophobic pocket, or both. Additionally, recombinant M295D/S363I and M295D/S363V double-mutant enzymes also seemed to have significantly higher K_o_ values and the activity retention profiles post-oxygen exposure seemed to indicate that the substitutions have an additive effect in both the enzymes. Not only were the observed K_o_ values 84-fold and 18-fold higher, respectively, than the wild-type enzyme, but the K_o_ of 427 μM for the M295D/S363I enzyme falls within the range of K_o_ values observed for many form I and form II RubisCOs [1,3]. Despite the increased K_o_ values, both the double-mutant enzymes had significantly lower *k*_cat_ values (Table 2). In summary, the results presented here (Tables 2 and 3) clearly indicate that Ser-363 is an influential site for reducing the *A*. *fulgidus* RbcL2 enzyme’s sensitivity to oxygen.

### In vivo complementation studies

The in vitro results were further supported by growth complementation studies with these altered enzymes under phototrophic conditions using the *R*. *capsulatus* SBI/II^-^ system. Under photoheterotrophic growth conditions, the double and triple mutant enzymes took considerably longer to support growth of the host organism compared to the wild-type and single mutant enzymes. Such results could be attributed to the extremely low *k*_cat_ and/or poor binding of RuBP by these enzymes. Nonetheless, mutant and wild-type enzymes were synthesized, as verified through SDS-PAGE gels, Western immunoblot analysis, and enzymatic activity assays at optimal temperatures for the *A*. *fulgidus* RbcL2 enzyme (83°C) after cultures were grown at the optimal growth temperature for *R*. *capsulatus* (30°C). Under photoautotrophic conditions, only the wild-type and single mutant forms of the enzyme were able to complement growth and it appeared that the mutant enzymes supported growth with shorter lag times compared to the wild type enzyme. It is tempting to attribute these differences to the ability of the mutant proteins to better cope with the small amounts of oxygen that might be present in the growth apparatus during the initial phases of the growth experiment, but this needs to be more fully established. Ultimately, because the single-mutant enzymes (M295D, S363I and S363V) are able to retain activity in vitro in the presence of oxygen, it should be possible for them to complement growth of *R*. *capsulatus* SBI/II- under aerobic chemoautotrophic growth conditions in the dark [23].

### Studies with the related *T*. *kodokaraensis* enzyme

Because these two highly related form III RubisCO enzymes share many similar features (39), it was difficult to conceive that the reported CO_2_/O_2_ substrate specificity (Ω) values for the two enzymes would be at such polar extremes when assayed at or near their temperature optimum (17, 20). Recombinant *T*. *kodakaraensis* RbcL was thus prepared and specificity values were determined under rigorously defined conditions with known concentrations of CO_2_ and O_2_. Our results indicate that the CO_2_/O_2_ substrate specificity value for *T*. *kodakaraensis* RbcL was 6 ± 0.2 at 83 C, which is much lower than the previously reported values of 290 and 310 at 80 C and 90 C, respectively [20] and more close to the value of 4 reported for the *A*. *fulgidus* enzyme at 83 C [17]. Moreover, we find that the *T*. *kodakaraensis* RbcL RubisCO is also oxygen sensitive, although not so sensitive as the closely related *A*. *fulgidus* RbcL2. Since the model structure *of A*. *fulgidus* RbcL2 is based on the solved structure of the highly homologous *T*. *kodakaraensis* enzyme, it is not surprising that many of the ionic bonding interactions that are suggested in the model structure of *A*. *fulgidus* RbcL2 appear in the solved structure of *T*. *kodakaraensis* RbcL [21]. Like the large subunits of all RubisCOs, known residues necessary for catalysis [11] are conserved and are positioned within the *T*. *kodakaraensis* structure in the same locale as in other RubisCO structures (S8 Fig). Similar to the model structure for *A*. *fulgidus* RbcL2, Met-298 in *T*. *kodakaraensis* RbcL was found to be situated on α–helix 5 positioned next to β–strand 5, adjacent to the active site. Met-298 was also found to be in close proximity to a highly conserved residue, Arg-282, found in all other forms of RubisCO and known to be necessary for substrate (RuBP) binding [34]. In *T*. *kodakaraensis* RbcL, there is no hydrogen bond to the Arg-282 residue, while there is definite hydrogen-bonding to the equivalent arginine residue in all other form I and form II RubisCO structures; e.g., originating from the oxygen atom of the carbonyl group of His-324 from the peptide backbone of the *Synechococcus* PCC6301 enzyme (S8 Fig). The distance between the corresponding arginine residue (Arg-282) to the carbonyl group of the equivalent histidine (His-311) of the peptide backbone is ~3.6 Å. It appears that an Asp substitution at position 298 would introduce an ionic interaction between this residue and Arg-282 (S9 Fig). Molecular modeling suggested that all the other amino acid substitutions made at position 298 would either result in unfavorable conformations or at the very least abrogate any possible interactions with Arg-282. In addition, many rotamers were available for the aspartic acid substitution at the methionine position; the rotamers with the lowest score, i.e., corresponding to the most favorable conformations, all showed the existence of possible hydrogen bonding interactions with Arg-282.

**Fig 6:**
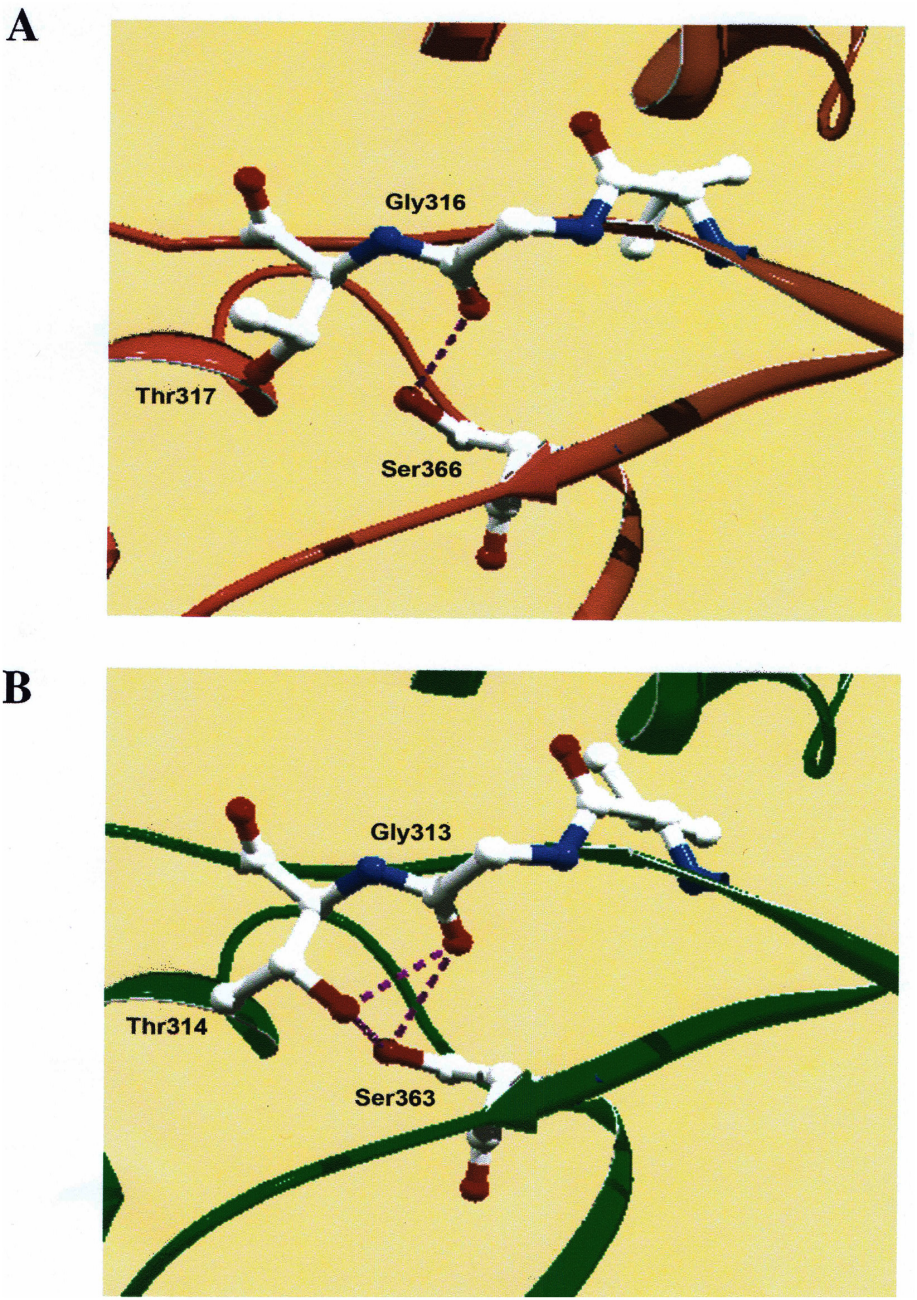
Comparison of side chain interactions of Ser-366 with Gly-316 and Thr-317 in the solved crystal structure of *T*. *kodakaraensis* RbcL with corresponding residues in the model structure of *A*. *fulgidus* RbcL2. Ser-366 in *T*. *kodakaraensis* RbcL (A) is situated on *β*-strand 6 pointing away from the active site, situated in a highly conserved hydrophobic pocket, and interacts with a highly conserved residue, Gly-316, depicted by dashed purple lines. Ser-366 does not interact with the other highly conserved residue, Thr-317. Conversely, the model structure of *A*. *fulgidus* RbcL2 (B) suggests that the identical residues in this region, Ser-363, Gly-313 and Thr-314 all interact to form hydrogen bonds (dashed purple lines).

Further investigation led us to another amino acid, Ser-366, which we predicted might have a similar effect on oxygen sensitivity since identical mutations were made in *A*. *fulgidus* RbcL2. Again, the structure indicated that this amino acid is on β-strand 6, situated in a hydrophobic pocket adjacent to the active site (Fig 6). Interestingly, when mutants S366I and S366V were generated, they both behaved similar to the equivalent *A*. *fulgidus* RbcL2 mutants. Also, the double-mutant enzymes of M298D/S366I and M298D/S366V showed an extremely low k_cat_ reminiscent of the values determined for the equivalent double mutants of *A*. *fulgidus* RbcL2. As previously explained, the model structure for *A*. *fulgidus* RbcL2 suggests that at this position, Ser-363, there is a van der Waals interaction with the main chain of Gly-313 and the side chain of Thr-314 that would conceivably be lost when either isoleucine or valine is introduced into the site (Fig 6). The solved structure for *T*. *kodakaraensis* RbcL shows a slightly different interaction. Compared to the *A*. *fulgidus* RbcL2 model, there is an interaction between the side chain of Ser-366 and the peptide backbone of Gly-316, however there is no interaction with the side chain of Thr-317 (Fig 6). Since the results with both *A*. *fulgidus* and *T*. *kodakaraensis* RubisCO mutant enzymes show similar trends with regard to changes in sensitivity to oxygen (as well as a substantial loss in activity for the *T*. *kodakaraensis* S366I enzyme), compared with the respective wild-type enzymes, perhaps the interaction that is most important is the interaction between the serine residue situated in the hydrophobic pocket and the highly conserved glycine residue. Perhaps the threonine residue does not affect the oxygen sensitivity as much, even though the model structure of *A*. *fulgidus* RbcL2 suggests that it does (Fig 6).

### The potential significance of a hydrophobic pocket

An important aspect of these studies is that residues identified, particularly Ser-363 of *A*. *fulgidus* RbcL2, reside in a hydrophobic pocket that is highly conserved in all three forms of RubisCO according to solved crystal and model structures [2,4–6,21,23,30,37,38,40,41]. Recent studies in our laboratory have shown that when alanine at position 375 (equivalent to Ser-363 of *A*. *fulgidus* RbcL2) was changed to a valine in the form I *Synechococcus* PCC6301 enzyme, the resultant A375V protein was almost completely oxygen insensitive and possessed a higher K_o_ compared to the wild-type enzyme [23]. Clearly, this localized hydrophobic pocket region of RubisCO influences oxygen interaction with the enediol intermediate of RuBP in the active site. This has now been confirmed with three different enzymes, the form I *Synechococcus* PCC6301 enzyme and the two archaeal form III enzymes, *A*. *fulgidus* RbcL2 and *T*. *kodakaraensis* RbcL, studied in this investigation. Upon further analysis, it is apparent that this hydrophobic pocket is located in a position that could influence the catalytically important loop-6 structure. Loop 6 folds over the active site after the insertion of RuBP into the active site; then a highly conserved lysine residue on loop 6 interacts with the N-terminal portion of the opposing large subunit to initiate the mechanistic steps that lead to product formation. Alterations to the hydrophobic pocket that is situated just below loop 6, specifically residues that reside within the pocket, such as Ser-363 in *A*. *fulgidus* RbcL2, Ser-366 in *T*. *kodakaraensis* RbcL, or Ala-375 in *Synechococcus* PCC6301 CbbL, could conceivably influence the ability of loop 6 to fold over the active site, interact with the N-terminal portion of the opposing subunit, and subsequently allow catalysis to occur. It is still unknown, but should continue to be investigated further, whether substitutions of either serine or alanine residues with bulky amino acids in the hydrophobic pocket of form III or form I enzymes, respectively, alter or distort the hydrophobic pocket as hypothesized. Whether such changes lead to alterations in loop 6 folding also should be further investigated as well as other residues of the hydrophobic region which might impact loop 6. In addition, it is interesting that the *T*. *kodakaraensis* archaeal enzyme substrate specificities are 6 at both high (83 C, reported here) and low (ambient) temperatures [22], which is different from the behavior of other nonthermophilic RubisCOs which show a decrease in specificity as the temperature increases up to 40 C [42,43]. The reason for these differences is not apparent but may be related to special properties conferred to the archaeal enzyme by virtue of its unique stability and its activation at high and low temperature extremes.

Recently, Tcherkez [44] has emphasized that there is a general lack of knowledge of the oxygenase reaction mechanism and the precise events leading to RubisCO interactions with this gaseous substrate. One recent investigation attempted to discern residues associated with binding of O_2_ and CO_2_ to the form I *Galdieria sulphuraria* enzyme [45], however crystallization of O_2_ bound enzyme was with inhibited (nitrosylated) and nonactivated (noncarbamylated) forms of the enzyme. This is tenuous as several previous studies have shown that the conformation of active-site residues in the unactivated and activated enzyme are quite different [11,20,40]. Rather, close scrutiny of residues that influence key kinetic properties as well as other mechanistic studies [42] where quantitative data can be related to protein structure may be a more productive approach towards understanding how RubisCO interacts with its gaseous substrates.

Although the role of the aforementioned amino acid residues is not precisely understood (specifically in the form III RubisCO enzymes studied here), the one form III structure available, combined with recent results, suggest a priority for more detailed structure/function studies; e.g., both wild-type and mutant forms of *A fulgidus* RbcL2 should be crystallized so that more direct observations can be made towards elucidating which precise changes occur to induce the rather substantial alterations in kinetic parameters. Such studies could also eventually lead to a suggested mechanism as to how the two gaseous substrates, carbon dioxide or oxygen, are differentiated in the overall mechanism in these RubisCO enzymes.

## Supporting Information

S1 Fig Far UV CD spectra of *A*. *fulgidus* RbcL2 wild-type and various mutant proteins under strict anaerobic conditions. All samples were first prepared under strict anaerobic conditions in the anaerobic chamber and were placed in a 1 cm path length quartz cuvette with screw cap lid and rubber septa for CD spectral determinations. Measurements were performed at 83°C as described in Materials and Methods. Wild-type (●), M295D (○), S363I (▼), S363V (Δ), M295D/S363I (■) and M295D/S363V (□) samples were analyzed in 20 mM Tris-HCl at a protein concentration of ~1 mg/ml.
(DOCX)

S2 Fig Far UV CD spectra of wild-type *A*. *fulgidus* RbcL2 under anaerobic and oxygen exposed conditions. Measurements were performed at 83°C as described in Materials and Methods. Anaerobic wild-type enzymes (●) was prepared anaerobically and measured in a quartz cuvette with a screw cap containing a rubber septa. The cuvette was then sparged with 100% oxygen and scanned (○). Samples were analyzed in 20 mM Tris-HCl at a protein concentration of ~1 mg/ml.
(DOCX)

S3 Fig Coomassie-stained discontinuous nondenaturing PAGE of samples of *A*. *fulgidus* RbcL2 wild-type and mutant RubisCOs prepared under anaerobic conditions and run under aerobic conditions. The *A*. *fulgidus rbcL2* gene was expressed in *E*. *coli* and samples were obtained either through partially purified heat stable extracts (where indicated) or FPLC column chromatography purification. 5 *μ*g of each sample was loaded per lane as follows: *A*. *fulgidus* wild-type (lane 1); M295D (lane 2); S363I (lane 3); heat stable extract I312S (lane 4); M295D/S363I (lane 5); heat stable extract M295D/I312S/S363I (lane 6); Native protein standard (lane 7).
(DOCX)

S4 Fig Coomassie-stained SDS-PAGE and Western immunoblot of extracts of photoheterotrophically-grown *R*. *capsulatus* SBI/II^-^ complemented with plasmid pRPS-MCS3-AfulRbcL2 (containing *A*. *fulgidus rbcL2*). The immunoblot was tested using antibodies directed against purified recombinant *A*. *fulgidus* RbcL2 RubisCO. All lanes contained soluble crude extract prepared from photoheterotrophically-grown stationary phase cultures from the following: wild-type *R*. *capsulatus* strain SB1003 (lane 2); wild-type *R*. *capsulatus* SBI/II^-^ containing pRPS-MCS3 with no insert (lane 3); *R*. *capsulatus* SBI/II^-^ complemented with plasmid pRPS-MCS3-MaceRbcL (containing *M*. *acetivorans rbcL*) (lane 4); *R*. *capsulatus* SBI/II^-^ complemented with plasmid pRPS-MCS3-AfulRbcL2 (containing *A*. *fulgidus rbcL2*) (lane 5); *R*. *capsulatus* SBI/II^-^ complemented with plasmid pRPS-MCS3-AfulRbcL2 mutated to M295D (lane 6); S363I (lane 7); S363V (lane 8); M295D/S363I (lane 9); M295D/S363V (lane 10); M295D/I312A/S363V (lane 11); M295D/I312S/S363V (lane 12); and purified recombinant *A*. *fulgidus* Rubisco (lane 13). Each lane received approximately 2 *μ*g of protein. BioRad Low Range Molecular Weight Standard was used as the marker in lane 1.
(DOCX)

S5 Fig Coomassie-stained SDS-PAGE (top) and Western immunoblot (bottom) of extracts of photoautotrophically-growwn *R*. *capsulatus* SBI/II^-^ complemented with plasmid pRPS-MCS3-AfulRbcL2 (containing *A*. *fulgidus rbcL2*). The immunoblot was tested using antibodies directed against purified recombinant *A*. *fulgidus* RbcL2 Rubisco. All lanes contained soluble crude extract prepared from stationary phase cultures grown photoautotrophically from the following: wild-type *R*. *capsulatus* strain SB1003 (lane 2); *R*. *capsulatus* SBI/II^-^ complemented with plasmid pRPS-MCS3-AfulRbcL2 (containing *A*. *fulgidus rbcL2*) (lane 3); *R*. *capsulatus* SBI/II^-^ complemented with plasmid pRPS-MCS3-AfulRbcL2 mutated to M295D (lane 4); S363I (lane 5); S363V (lane 6). Each lane received approximately 2 *μ*g of protein. BioRad Low Range Molecular Weight Standard was used as the marker in lane 1.
(DOCX)

S6 Fig Coomassie-stained SDS-PAGE of samples from *T*. *kodakaraensis* RbcL RubisCO purification. The *T*. *kodakaraensis rbcL* gene was expressed in *E*. *coli* and samples obtained from: uninduced *E*. *coli* cells (lane 2); soluble extract of French Press disrupted *E*. *coli* cells after induction (lane 3); supernatant obtained after centrifuging the heat-treated (90°C) extract for 20 min (lane 4); Q-Sepharose anion exchange chromatography (lane 5); Superose-12 gel filtration (lane 6); phenyl-Sepharose hydrophobic chromatography (lane 7). Lane 1 contains low range SDS protein standards.
(DOCX)

S7 Fig Coomassie-stained native PAGE of samples from *A*. *fulgidus* RbcL2 and *T*. *kodakaraensis* RbcL RubisCOs. Native PAGE protein standards (lane 1); *A*. *fulgidus* RbcL2 (lane 2); *T*. *kodakaraensis* RbcL (lane 3).
(DOCX)

S8 Fig Comparison of side-chain interactions with Arg-295 in form I *Synechococcus* PCC6301 and Arg-282 and in form III *T*. *kodakaraensis* RubisCO enzymes. Highly conserved amino acids necessary for the binding of the five carbon substrate, RuBP, in *Synechococcus* PCC6301/*T*. *kodakaraensis* enzymes include Arg-295/Arg-282, His-298/His-285, and His-327/His-314 and are shown in ball and stick figures off of the ribbon structure. Phe-311 in *Synechococcus* PCC6301 and Met-298 in *T*. *kodakaraensis* are at the same position in sequence alignments, situated on *α*-helix 5. The carbonyl of the peptide backbone of His-327 in *Synechococcus* PCC6301 Rubisco forms an ionic interaction (depicted by a dashed purple line) with the side chain of Arg-295 (A) whereas this interaction is not observed between the corresponding His-314 and Arg-282 in *T*. *kodakaraensis* Rubisco (B).
(DOCX)

S9 Fig Predicted side-chain interactions with Met-298 in wild-type *T*. *kodakaraensis* RbcL and the mutant M298D enzyme. Side chains shown are amino acids Met-298 (A) and Asp-298 (B), as well as conserved amino acids found in all other forms of RubisCO. In *T*. *kodakaraensis* RbcL and the mutant M298D enzyme, His-285, Arg-282, and His-314, are illustrated as they are necessary for catalysis and binding of the five carbon substrate, RuBP. The solved crystal structure shows no ionic interactions between Arg-282 and Met-298 in the wild-type form of the enzyme (A). In the M298D mutant, the model predicts an ionic interaction between the hydroxyl group of the Asp-298 residue and the amino group of the Arg-282 residue (dashed purple line).
(DOCX)

S1 Table Plasmids and strains used in this study. (DOCX)

S2 Table Carboxylase activity at 83°C of heat stable extracts of *E*. *coli* containing *A*. *fulgidus* RbcL2 wild type and mutant enzymes under anaerobic and oxygen exposed conditions. (DOCX)

S3 Table Specific activity of crude soluble wild-type and mutant *A*. *fulgidus* RbcL2 obtained from *R*. *capsulatus* SBI/II^-^ grown photoheterotrophically and photoautotrophically. (DOCX)
