# Supplementary material for: Serine 363 of a Hydrophobic Region of Archaeal Ribulose 1,5-Bisphosphate Carboxylase/Oxygenase from Archaeoglobus fulgidus and Thermococcus kodakaraensis Affects CO2/O2 Substrate Specificity and Oxygen Sensitivity
Source: PLoS One. 2015 Sep 18;10(9):e0138351. doi: 10.1371/journal.pone.0138351 (PMC4575112; doi:10.1371/journal.pone.0138351)
Supplement: S1 Fig — All samples were first prepared under strict anaerobic conditions in the anaerobic chamber and were placed in a 1 cm path length quartz cuvette with screw cap lid and rubber septa for CD spectral determinations. Measurements were performed at 83°C as described in Materials and Methods. Wild-type (●), M295D (○), S363I (▼), S363V (Δ), M295D/S363I (■) and M295D/S363V (□) samples were analyzed in 20 mM Tris-HCl at a protein concentration of ~1 mg/ml. (DOCX) [file pone.0138351.s001.docx]

**S1 Fig. Far UV CD spectra of *A. fulgidus* RbcL2 wild-type and various mutant proteins under strict anaerobic conditions.**
