# Supplementary material for: Serine 363 of a Hydrophobic Region of Archaeal Ribulose 1,5-Bisphosphate Carboxylase/Oxygenase from Archaeoglobus fulgidus and Thermococcus kodakaraensis Affects CO2/O2 Substrate Specificity and Oxygen Sensitivity
Source: PLoS One. 2015 Sep 18;10(9):e0138351. doi: 10.1371/journal.pone.0138351 (PMC4575112; doi:10.1371/journal.pone.0138351)
Supplement: S2 Fig — Measurements were performed at 83°C as described in Materials and Methods. Anaerobic wild-type enzymes (●) was prepared anaerobically and measured in a quartz cuvette with a screw cap containing a rubber septa. The cuvette was then sparged with 100% oxygen and scanned (○). Samples were analyzed in 20 mM Tris-HCl at a protein concentration of ~1 mg/ml. (DOCX) [file pone.0138351.s002.docx]

**S2 Fig. Far UV CD spectra of wild-type *A. fulgidus* RbcL2 under anaerobic and oxygen exposed conditions.**

**S2 Fig. Far UV CD spectra of wild-type *A. fulgidus* RbcL2 under anaerobic and oxygen exposed conditions.**
