# Supplementary material for: Serine 363 of a Hydrophobic Region of Archaeal Ribulose 1,5-Bisphosphate Carboxylase/Oxygenase from Archaeoglobus fulgidus and Thermococcus kodakaraensis Affects CO2/O2 Substrate Specificity and Oxygen Sensitivity
Source: PLoS One. 2015 Sep 18;10(9):e0138351. doi: 10.1371/journal.pone.0138351 (PMC4575112; doi:10.1371/journal.pone.0138351)
Supplement: S3 Fig — The A. fulgidus rbcL2 gene was expressed in E. coli and samples were obtained either through partially purified heat stable extracts (where indicated) or FPLC column chromatography purification. 5 μg of each sample was loaded per lane as follows: A. fulgidus wild-type (lane 1); M295D (lane 2); S363I (lane 3); heat stable extract I312S (lane 4); M295D/S363I (lane 5); heat stable extract M295D/I312S/S363I (lane 6); Native protein standard (lane 7). (DOCX) [file pone.0138351.s003.docx]

**S3 Fig. Coomassie-stained discontinuous nondenaturing PAGE of samples of *A. fulgidus* RbcL2 wild-type and mutant RubisCOs prepared under anaerobic conditions and run under aerobic conditions.**
