# Supplementary material for: Serine 363 of a Hydrophobic Region of Archaeal Ribulose 1,5-Bisphosphate Carboxylase/Oxygenase from Archaeoglobus fulgidus and Thermococcus kodakaraensis Affects CO2/O2 Substrate Specificity and Oxygen Sensitivity
Source: PLoS One. 2015 Sep 18;10(9):e0138351. doi: 10.1371/journal.pone.0138351 (PMC4575112; doi:10.1371/journal.pone.0138351)
Supplement: S4 Fig — The immunoblot was tested using antibodies directed against purified recombinant A. fulgidus RbcL2 RubisCO. All lanes contained soluble crude extract prepared from photoheterotrophically-grown stationary phase cultures from the following: wild-type R. capsulatus strain SB1003 (lane 2); wild-type R. capsulatus SBI/II- containing pRPS-MCS3 with no insert (lane 3); R. capsulatus SBI/II- complemented with plasmid pRPS-MCS3-MaceRbcL (containing M. acetivorans rbcL) (lane 4); R. capsulatus SBI/II- complemented with plasmid pRPS-MCS3-AfulRbcL2 (containing A. fulgidus rbcL2) (lane 5); R. capsulatus SBI/II- complemented with plasmid pRPS-MCS3-AfulRbcL2 mutated to M295D (lane 6); S363I (lane 7); S363V (lane 8); M295D/S363I (lane 9); M295D/S363V (lane 10); M295D/I312A/S363V (lane 11); M295D/I312S/S363V (lane 12); and purified recombinant A. fulgidus Rubisco (lane 13). Each lane received approximately 2 μg of protein. BioRad Low Range Molecular Weight Standard was used as the marker in lane 1. (DOCX) [file pone.0138351.s004.docx]

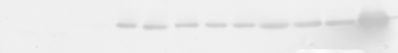


**1**

**2**

**3**

**4**

**5**

**6**

**8**

**7**

**10**

**11**

**9**

**12**

**13**

**RbcL2**

**RbcL2**

**S4 Fig. Coomassie-stained SDS-PAGE (top) and Western immunoblot (bottom) of extracts of photoheterotrophically-grown *R. capsulatus* SBI/II^-^ complemented with plasmid pRPS-MCS3-AfulRbcL2 (containing *A. fulgidus rbcL2*).**
