# Supplementary material for: Serine 363 of a Hydrophobic Region of Archaeal Ribulose 1,5-Bisphosphate Carboxylase/Oxygenase from Archaeoglobus fulgidus and Thermococcus kodakaraensis Affects CO2/O2 Substrate Specificity and Oxygen Sensitivity
Source: PLoS One. 2015 Sep 18;10(9):e0138351. doi: 10.1371/journal.pone.0138351 (PMC4575112; doi:10.1371/journal.pone.0138351)
Supplement: S5 Fig — The immunoblot was tested using antibodies directed against purified recombinant A. fulgidus RbcL2 Rubisco. All lanes contained soluble crude extract prepared from stationary phase cultures grown photoautotrophically from the following: wild-type R. capsulatus strain SB1003 (lane 2); R. capsulatus SBI/II- complemented with plasmid pRPS-MCS3-AfulRbcL2 (containing A. fulgidus rbcL2) (lane 3); R. capsulatus SBI/II- complemented with plasmid pRPS-MCS3-AfulRbcL2 mutated to M295D (lane 4); S363I (lane 5); S363V (lane 6). Each lane received approximately 2 μg of protein. BioRad Low Range Molecular Weight Standard was used as the marker in lane 1. (DOCX) [file pone.0138351.s005.docx]

**RbcL2**

**RbcL2**

**1 2 3 4 5 6**

**S5 Fig. Coomassie-stained SDS-PAGE (top) and Western immunoblot (bottom) of extracts of photoautotrophically-grown *R. capsulatus* SBI/II^-^ complemented with plasmid pRPS-MCS3-AfulRbcL2 (containing *A. fulgidus rbcL2*).**
