# Supplementary material for: Serine 363 of a Hydrophobic Region of Archaeal Ribulose 1,5-Bisphosphate Carboxylase/Oxygenase from Archaeoglobus fulgidus and Thermococcus kodakaraensis Affects CO2/O2 Substrate Specificity and Oxygen Sensitivity
Source: PLoS One. 2015 Sep 18;10(9):e0138351. doi: 10.1371/journal.pone.0138351 (PMC4575112; doi:10.1371/journal.pone.0138351)
Supplement: S6 Fig — The T. kodakaraensis rbcL gene was expressed in E. coli and samples obtained from: uninduced E. coli cells (lane 2); soluble extract of French Press disrupted E. coli cells after induction (lane 3); supernatant obtained after centrifuging the heat-treated (90°C) extract for 20 min (lane 4); Q-Sepharose anion exchange chromatography (lane 5); Superose-12 gel filtration (lane 6); phenyl-Sepharose hydrophobic chromatography (lane 7). Lane 1 contains low range SDS protein standards. (DOCX) [file pone.0138351.s006.docx]

**S6 Fig. Coomassie-stained SDS-PAGE of samples from *T. kodakaraensis* RbcL RubisCO purification.**
