# Supplementary material for: Serine 363 of a Hydrophobic Region of Archaeal Ribulose 1,5-Bisphosphate Carboxylase/Oxygenase from Archaeoglobus fulgidus and Thermococcus kodakaraensis Affects CO2/O2 Substrate Specificity and Oxygen Sensitivity
Source: PLoS One. 2015 Sep 18;10(9):e0138351. doi: 10.1371/journal.pone.0138351 (PMC4575112; doi:10.1371/journal.pone.0138351)
Supplement: S7 Fig — Native PAGE protein standards (lane 1); A. fulgidus RbcL2 (lane 2); T. kodakaraensis RbcL (lane 3). (DOCX) [file pone.0138351.s007.docx]

**S7 Fig. Coomassie-stained native PAGE of samples from *A. fulgidus* RbcL2 and *T. kodakaraensis* RbcL RubisCOs.**
