# Supplementary material for: Serine 363 of a Hydrophobic Region of Archaeal Ribulose 1,5-Bisphosphate Carboxylase/Oxygenase from Archaeoglobus fulgidus and Thermococcus kodakaraensis Affects CO2/O2 Substrate Specificity and Oxygen Sensitivity
Source: PLoS One. 2015 Sep 18;10(9):e0138351. doi: 10.1371/journal.pone.0138351 (PMC4575112; doi:10.1371/journal.pone.0138351)
Supplement: S8 Fig — Highly conserved amino acids necessary for the binding of the five carbon substrate, RuBP, in Synechococcus PCC6301/T. kodakaraensis enzymes include Arg-295/Arg-282, His-298/His-285, and His-327/His-314 and are shown in ball and stick figures off of the ribbon structure. Phe-311 in Synechococcus PCC6301 and Met-298 in T. kodakaraensis are at the same position in sequence alignments, situated on α-helix 5. The carbonyl of the peptide backbone of His-327 in Synechococcus PCC6301 Rubisco forms an ionic interaction (depicted by a dashed purple line) with the side chain of Arg-295 (A) whereas this interaction is not observed between the corresponding His-314 and Arg-282 in T. kodakaraensis Rubisco (B). (DOCX) [file pone.0138351.s008.docx]

**A**

**B**

**S8 Fig. Comparison of side-chain interactions with Arg-295 in (A) form I *Synechococcus* PCC6301 and Arg-282 and in (B) form III *T. kodakaraensis* RubisCO enzymes.**
