# Supplementary material for: Serine 363 of a Hydrophobic Region of Archaeal Ribulose 1,5-Bisphosphate Carboxylase/Oxygenase from Archaeoglobus fulgidus and Thermococcus kodakaraensis Affects CO2/O2 Substrate Specificity and Oxygen Sensitivity
Source: PLoS One. 2015 Sep 18;10(9):e0138351. doi: 10.1371/journal.pone.0138351 (PMC4575112; doi:10.1371/journal.pone.0138351)
Supplement: S9 Fig — Side chains shown are amino acids Met-298 (A) and Asp-298 (B), as well as conserved amino acids found in all other forms of RubisCO. In T. kodakaraensis RbcL and the mutant M298D enzyme, His-285, Arg-282, and His-314, are illustrated as they are necessary for catalysis and binding of the five carbon substrate, RuBP. The solved crystal structure shows no ionic interactions between Arg-282 and Met-298 in the wild-type form of the enzyme (A). In the M298D mutant, the model predicts an ionic interaction between the hydroxyl group of the Asp-298 residue and the amino group of the Arg-282 residue (dashed purple line). (DOCX) [file pone.0138351.s009.docx]

**A**

**B**

**S9 Fig. Predicted side-chain interactions with Met-298 in wild-type *T. kodakaraensis* RbcL and the mutant M298D enzyme.**
