# Supplementary material for: Serine 363 of a Hydrophobic Region of Archaeal Ribulose 1,5-Bisphosphate Carboxylase/Oxygenase from Archaeoglobus fulgidus and Thermococcus kodakaraensis Affects CO2/O2 Substrate Specificity and Oxygen Sensitivity
Source: PLoS One. 2015 Sep 18;10(9):e0138351. doi: 10.1371/journal.pone.0138351 (PMC4575112; doi:10.1371/journal.pone.0138351)
Supplement: S1 Table — (DOCX) [file pone.0138351.s010.docx]

**S1 Table. Plasmids and strains used in this study.**

| Plasmid or Strain | Relevant Characteristics | Reference |
| --- | --- | --- |
| Plasmids |  |  |
| pET11a | pBR322 derivative containing T7 autogenes | 1 |
| pET11a-AfulRbcL2 | pET11a with *NdeI*/*BamHI - Af 1638* | 2 |
| pET11a-TkodRbcL | pET11a with *NdeI*/*BamHI - Tk 2290* | This study |
| pRPS-MCS3 | Broad host range vector derivative from  pBBR1-MCS3 containing p*cbbM* and *cbbR* | 3 |
| pRPS-MCS3-AfulRbcL2 | pRPS-MCS3 with *KpnI*/*SacI - Af 1638* | This study |
| pCR**-**2.1-TOPO-  TkodRbcL | pCR-2.1-TOPO with PCR product *Tk 2290* | This study |
| pRK2013 | Harbors transfer genes required for conjugation | 4 |
| Bacterial Strains |  |  |
| *E. coli* JM109 | Appropriate strain used for routine cloning  and high quality miniprep DNA applications | 5 |
| *E. coli* BL21(DE3) | High-level expression by IPTG induction of T7 RNA polymerase from *lacUV5* promoter | 6 |
| *E. coli* XL-10 Gold | Possesses phenotype which increases transformation efficiency of DNA used in site-directed mutagenesis. | 7 |
| *E. coli* HB101 | Host strain harboring pRK2013 | 8 |
| *R. capsulatus* SB1003 | Wild-type | 9 |
| *R. capsulatus* SBI/II^-^ | *cbbLS/ cbbM* | 10 |
