# Supplementary material for: Serine 363 of a Hydrophobic Region of Archaeal Ribulose 1,5-Bisphosphate Carboxylase/Oxygenase from Archaeoglobus fulgidus and Thermococcus kodakaraensis Affects CO2/O2 Substrate Specificity and Oxygen Sensitivity
Source: PLoS One. 2015 Sep 18;10(9):e0138351. doi: 10.1371/journal.pone.0138351 (PMC4575112; doi:10.1371/journal.pone.0138351)
Supplement: S2 Table — (DOCX) [file pone.0138351.s011.docx]

**S2 Table. Carboxylase activity at 83°C of heat stable extracts of *E. coli* containing *A. fulgidus* RbcL2 wild type and mutant enzymes under anaerobic and oxygen exposed conditions.**

| Enzymes | Anaerobic^a^ | O_2_-exposed^a^ | % activity retained |
| --- | --- | --- | --- |
| Wild-type | 1.234 | 0.251 | 20 |
| M295D | 1.825 | 0.796 | 44 |
| S363I | 1.769 | 1.345 | 76 |
| S363V | 2.543 | 1.158 | 46 |
| I312A | 0.957 | 0.284 | 30 |
| I312S | 1.323 | 0.208 | 16 |
| I312T | 1.503 | 0.230 | 15 |
| M295D/S363I | 0.087 | 0.081 | 93 |
| M295D/S363V | 0.248 | 0.224 | 90 |
| M295D/I312A | 1.297 | 0.523 | 40 |
| M295D/I312S | 1.109 | 0.507 | 46 |
| M295D/I312T | 2.246 | 1.003 | 45 |
| I312A/S363I | 1.488 | 1.117 | 75 |
| I312S/S363I | 1.197 | o.943 | 78 |
| M295D/I312A/S363I | 0.051 | 0.046 | 89 |
| M295D/I312S/S363I | 0.046 | 0.040 | 87 |
| M295D/I312A/S363V | 0.146 | 0.103 | 70 |
| M295D/I312S/S363V | 0.140 | 0.104 | 74 |

^a^ Average of duplicate assays with results expressed in µmol CO_2_ fixed/min/mg protein. Enzymes were exposed to oxygen for a minimum of 10 min as described in Materials and Methods.
