# Supplementary material for: Serine 363 of a Hydrophobic Region of Archaeal Ribulose 1,5-Bisphosphate Carboxylase/Oxygenase from Archaeoglobus fulgidus and Thermococcus kodakaraensis Affects CO2/O2 Substrate Specificity and Oxygen Sensitivity
Source: PLoS One. 2015 Sep 18;10(9):e0138351. doi: 10.1371/journal.pone.0138351 (PMC4575112; doi:10.1371/journal.pone.0138351)
Supplement: S3 Table — (DOCX) [file pone.0138351.s012.docx]

**S3 Table. Specific activity of crude soluble wild-type and mutant *A. fulgidus* RbcL2 obtained from *R. capsulatus* SBI/II^-^ grown photoheterotrophically and photoautotrophically**

| Enzymes | Photoheterotrophic | | Photoautotrophic | |
| --- | --- | --- | --- | --- |
|  | 30°C^a^ | 83°C^a^ | 30°C^a^ | 83°C^a^ |
| Wild-type | 14 | 353 | 226 | 3797 |
| M295D | 10 | 218 | 129 | 1805 |
| S363I | 0.2 | 22 | 86 | 1237 |
| S363V | 9 | 566 | 93 | 1896 |
| M295D/S363I | 0.5 | 60 | NG | NG |
| M295D/S363V | 1.2 | 268 | NG | NG |
| M295D/I312A/S363V | ND | 11 | NG | NG |
| M295D/I312S/S363V | 0.2 | 20 | NG | NG |

^a^ Average of duplicate assays with results expressed in nmol CO_2_ fixed/min/mg; ND represents assays where no activity was detected; NG represents samples that were unable to grow under the specific growth condition, thus assays were unable to be performed.
